# Advances in the study of marketed antibody-drug Conjugates (ADCs) for the treatment of breast cancer

**DOI:** 10.3389/fphar.2023.1332539

**Published:** 2024-01-30

**Authors:** Yan Liang, Purong Zhang, Feng Li, Houyun Lai, Tingting Qi, Yixin Wang

**Affiliations:** ^1^ Sichuan Cancer Hospital, Cancer Hospital Affiliate University of Electronic Science and Technology, Chengdu, China; ^2^ School of Medicine, University of Electronic Science and Technology, Chengdu, China

**Keywords:** breast cancer, T-DM1, T-DXd, SG, ADC

## Abstract

Breast cancer continues to have a high incidence rate among female malignancies. Despite significant advancements in treatment modalities, the heterogeneous nature of breast cancer and its resistance to various therapeutic approaches pose considerable challenges. Antibody-drug conjugates (ADCs) effectively merge the specificity of antibodies with the cytotoxicity of chemotherapeutic agents, offering a novel strategy for precision treatment of breast cancer. Notably, trastuzumab emtansine (T-DM1) has provided a new therapeutic option for HER2-positive breast cancer patients globally, especially those resistant to conventional treatments. The development of trastuzumab deruxtecan (T-DXd) and sacituzumab govitecan (SG) has further broadened the applicability of ADCs in breast cancer therapy, presenting new hopes for patients with low HER2 expression and triple-negative breast cancer. However, the application of ADCs presents certain challenges. For instance, their treatment may lead to adverse reactions such as interstitial lung disease, thrombocytopenia, and diarrhea. Moreover, prolonged treatment could result in ADCs resistance, complicating the therapeutic process. Economically, the high costs of ADCs might hinder their accessibility in low-income regions. This article reviews the structure, mechanism of action, and clinical trials of commercially available ADCs for breast cancer treatment, with a focus on the clinical trials of the three drugs, aiming to provide insights for clinical applications and future research.

## 1 Introduction

Breast cancer leads the incidence of female cancers, it ranks second in lethality after lung cancer, and the number of new cases is increasing every year (Sung et al., 2021; Giaquinto et al., 2022; Siegel et al., 2022). Breast cancer can be classified based on molecular markers such as estrogen receptors, progesterone receptors, and human epidermal growth factor 2 into three major subtypes: hormone receptor-positive/HER2-negative (accounting for approximately 70%), HER2-positive (comprising 15%–20%), and triple-negative (lacking all three standard molecular markers, representing 15%). The first-line treatment for the hormone receptor-positive/HER2-negative subtype is typically endocrine therapy, which has a favorable prognosis and a low recurrence rate, but may lead to hormone therapy resistance. HER2-positive subtypes primarily receive HER2-targeted antibodies or small molecule inhibitors in combination with chemotherapy, but may also subsequently develop targeted therapy resistance. Triple-negative breast cancer (TNBC) is especially prevalent among young women under 40 years old and, compared to other subtypes, has a worse prognosis, higher metastatic rate, and is more recurrent (Waks and Winer, 2019). Due to the high heterogeneity of TNBC, it is often insensitive to certain targeted drugs and immunotherapies. Standard chemotherapy agents, such as taxanes and anthracyclines, are the primary treatments, but their efficacy is suboptimal (Waks and Winer, 2019; Derakhshan and Reis-Filho, 2022).

Against this backdrop, the emergence of Antibody-Drug Conjugates (ADCs) represents a new direction in breast cancer treatment. ADCs mainly consist of three key components: 1) The antibody component, typically a monoclonal antibody (mAb) with high specificity; 2) The toxin drug component, a small molecular compound with potent cytotoxicity; and 3) The linker component, designed to be stable in the body but capable of releasing the drug under specific conditions. Trastuzumab emtansine (T-DM1), trastuzumab deruxtecan (T-DXd), and sacituzumab govitecan (SG) are the main ADCs available for treating breast cancer. T-DM1 is a combination of trastuzumab and the DM1 derivative; T-DXd comprises an anti-HER2 monoclonal antibody combined with the DXd derivative of irinotecan; sacituzumab govitecan is an ADC targeting the TROP-2 antigen, leveraging the high expression of TROP-2 in various tumors for its unique mechanism of action (Bardia et al., 2019; Goldenberg and Sharkey, 2020).

The application of ADCs not only provides new options for HER2-positive breast cancer treatment but also brings renewed hope for challenging cases like TNBC. By analyzing relevant literature and clinical trial data (updated to 15 October 2023), this article delves into the currently available ADCs for breast cancer treatment, encompassing their structure, mechanism, clinical applications, and future prospects, aiming to offer valuable insights for clinicians and researchers.

## 2 Antibody or target antigen

### 2.1 Antibodies and target antigens for T-DM1 and T-DXd:trastuzumab and HER2

The human epidermal growth factor receptor (HER) family comprises four tyrosine kinases, namely, EGFR (HER1, erbB1), HER2 (erbB2, HER2/neu), HER3 (erbB3), and HER4 (erbB4) (Soosanabadi et al., 2022). Although HER2 has no specific ligand, it is the primary dimerization partner for the other three receptors in the family. When these receptors undergo homo- or heterodimerization, they activate downstream signaling pathways such as MAPK, Ras/Raf and PI3K/AKT, which in turn affects key cellular functions such as proliferation, motility, survival, and adhesion (Nicolini et al., 2018; Miricescu et al., 2020). The oncogene of HER2, ERBB2, is located on chromosome 17q12.Amplification of HER2 is the primary cause of overexpression of its receptor and this amplification plays a plays a key role in tumor formation and development. Notably, HER2 expression is relatively low in normal tissues, but amplification of HER2 occurs in about 15%–20% of breast cancer patients (Krishnamurti and Silverman, 2014). In addition, certain breast cancers initially diagnosed as HR+/HER2-may switch from negative to positive HER2 after hormonal therapy (Chaudhary et al., 2023; Will et al., 2023). It has been shown that ER+/HER2 protein overexpressing ductal carcinoma *in situ* (DCIS), especially tumors with high ER, frequently overexpress HER2 protein without gene amplification, and tumor cells overexpressing HER2 protein corresponds to the microenvironment of the DCIS, which may be due to hypoxic status, whereas during progression to an invasive disease, the tumor microenvironment may undergo drastic changes with neovascularization including neovascularization Tissue components including stromal cells interacting with the stromal cells, thereby decreasing HER2 protein expression (Horimoto et al., 2019). On chromosome 17q12-q21, genes in proximity to the HER2/ERBB2 locus often undergo co-amplification with the HER2/ERBB2 gene. This phenomenon not only results in the overexpression of HER2 but also potentially amplifies the expression of adjacent genes, thereby elevating the complexity and invasiveness of the cancer (Moasser, 2007). While initial studies indicated a correlation between HER2/neu amplification and adverse prognosis and recurrence in breast cancer patients (Slamon et al., 1987), guidelines set forth by the American Society of Clinical Oncology (ASCO) underscore that HER2 status should primarily guide the selection of suitable treatment modalities rather than serve as a prognostic indicator. Lastly, in breast cancer, there’s a frequent co-amplification of HER2 and TOP2A genes (Wolff et al., 2018). New study shows that breast cancer patients whose tumors express HER2 protein without HER2 gene amplification (HER2-low) can benefit from antibody-drug conjugates (ADCs) (Modi et al., 2022a).

In the antibody-drug conjugates (ADCs) T-DM1 and T-DXd, trastuzumab acts as the monoclonal antibody (mAB) component, specifically recognizing and binding to the HER2 protein overexpressed on the surface of cancer cells. On the one hand: trastuzumab inhibits tumor cell growth and differentiation by binding to the HER2 extracellular structural domain and inhibiting the MAPK and PI3K/Akt pathways (Junttila et al., 2009). On the other hand, trastuzumab also activates the internalization mechanism of the T-DC complex, which mediates endocytosis through the receptor, after which the ADC complex-containing endosome matures and fuses with the lysosome (von Arx et al., 2023). Additionally, trastuzumab can promote antibody-dependent cell-mediated cytotoxicity (ADCC) by immune cells against tumor cells, leading to their destruction (De et al., 2013). Previously, trastuzumab was mainly used alone or in combination with chemotherapeutic agents to treat HER2-positive breast cancer, but is now also used in neoadjuvant and adjuvant therapies aimed at shrinking the size of tumors or reducing the risk of recurrence (Swain et al., 2023). Nevertheless, a meta-analysis evaluating trastuzumab-specific-induced adverse reactions collected 25 statistically and clinically significant adverse reactions associated with trastuzumab, such as infusion-related fever and chills, cardiotoxicity, gastrointestinal distress, hematologic toxicity, increased risk of infection, and skin problems (Jackson et al., 2022). The presence of cardiotoxicity is an indicator for discontinuation of trastuzumab. The use of trastuzumab may increase the risk of cardiotoxicity in the setting of cardiovascular disease, advanced age, and prior or concurrent anthracycline therapy (Dempsey et al., 2021). Unlike anthracyclines, trastuzumab-induced cardiotoxicity is mostly asymptomatic, dose-independent, and largely reversible, and in some cases can be prevented or mitigated by antioxidant use (Méndez-Valdés et al., 2023).

### 2.2 Target antigens of SG: Trop-2

Trop-2, also recognized as human trophoblast epidermal cell antigen, tumor-associated calcium signal transducer 2 (TACSTD2), membrane component chromosome 1 surface marker 1 (M1S1), gastrointestinal antigen 733–1 (GA733-1), and epithelial glycoprotein-1 (EGP-1), is a cell surface glycoprotein (Goldenberg et al., 2018). The gene encoding Trop-2 is Tacstd2, which, while not a cancer gene itself, is correlated with cancer. It exhibits overexpression in a multitude of advanced epithelial cancers such as breast, ovarian, and colon cancers, relative to corresponding normal tissues (Stepan et al., 2011; Shvartsur and Bonavida, 2014). Trop-2 influences tumor-associated calcium signaling and heightens the activation of ERK/MAPK pathway signaling. Moreover, it modifies cell cycle protein D1/E levels, leading to an altered cell cycle and uncontrolled cell growth, which in turn impacts cancer cell proliferation, migration, invasion, and survival (Liu et al., 2022).

Trop-2 is widely overexpressed across various types of breast cancer. A study conducted by Merve Aslan and colleagues evaluated Trop2 protein levels in 75 ER+, HER2+, and TNBC samples, revealing that Trop-2 was overexpressed in 50% of ER+, 74% of HER2+, and 93% of TNBC cases (Aslan et al., 2021). Neelima Vidula and her team evaluated the correlation between Trop2 gene expression and clinical and tumor characteristics in primary breast cancer (BC) using several databases, including I-SPY 1 (*n* = 149), METABRIC (*n* = 1992), TCGA (*n* = 817), and the full sample of the Kruskal-Wallis trial. They concluded that Trop-2 gene expression was observed across all BC subtypes, particularly Luminal A and TNBC (Vidula et al., 2017). *In vivo* upregulation of Trop-2 can stimulate tumor growth, and Trop-2 protein levels are significantly elevated in breast cancer patients. Merve Aslan and colleagues reported a significant inhibition of TNBC cell growth, colony formation *in vitro*, and tumor growth *in vivo* following the downregulation of Trop-2 through gene deletion or gene silencing (Trerotola et al., 2013; Aslan et al., 2021). The TACSTD2 gene encodes the Trop-2 protein, and its high expression is correlated with a poor prognosis in breast cancer. Trop-2 can promote migration and invasion of BC cells by inducing epithelial-mesenchymal transition (EMT), leading to lymph node metastasis and distant metastasis. Additionally, studies have indicated that the prognostic outcome of Trop-2 differs based on its cellular location, with its presence in the cell membrane being unfavorable for prognosis and intracellular distribution being favorable (Lin et al., 2013; Ambrogi et al., 2014; Zhao et al., 2018; Jeon et al., 2022).

## 3 Cytotoxic drug (payload)

The payloads of antibody-coupled drug selection are mostly derivatives of some old drugs, and these former drugs are rarely used in breast cancer chemotherapy, and the relevant references are rather old. This part mainly introduces the general process of the derivation of these old drugs, as well as the related pharmacological effects and adverse reactions.

### 3.1 Payload of T-DM1: DM1, derivative of maytansine

Maytansine is the first ansa macrolide isolated from plants rather than being extracted from microorganisms (Kupchan et al., 1977). It shares a binding site with vinca alkaloids on microtubule proteins, thereby possessing the capability to target microtubules and interrupt the cell cycle (Bhattacharyya and Wolff, 1977). Early studies have shown that maytansine exhibits high activity against P388 lymphocytic leukemia in mice and also demonstrates significant inhibitory effects on L1210 mouse leukemia, Lewis lung carcinoma, and B-16 melanoma solid tumors (Issell and Crooke, 1978). However, subsequent clinical studies revealed that maytansine is associated with various adverse reactions, including gastrointestinal symptoms, central and peripheral nervous system issues, liver function abnormalities, bone marrow suppression, and phlebitis (Cassady et al., 2004). Due to its narrow therapeutic window and pronounced toxic side effects, maytansine, when used alone, has not been widely considered as an effective antitumor drug (Thigpen et al., 1983). After a period of research stagnation, the anticancer potential of maytansine regained attention in the scientific community. This resurgence can be attributed to the research team at ImmunoGen Inc., who not only successfully synthesized derivatives of maytansine but also found that these derivatives have cellular toxicity far exceeding that of traditional anticancer drugs, specifically, their toxicity is 100–1000 times that of conventional drugs. These newly synthesized maytansine derivatives are collectively referred to as “maytansinoids” or “DMs” (Chari et al., 1992). Among them, DM1, a semi-synthetic derivative of maytansine, can bind to microtubule proteins and, by inhibiting microtubule dynamics, halt the cell cycle at the G2/M phase, thereby suppressing cell division and inducing apoptosis. Compared to maytansine, DM1 exhibits higher cellular toxicity, making it an ideal toxin carrier for antibody-drug conjugates (ADCs) (Lopus, 2011).

### 3.2 Payload of T-DXd: DXd, derivative of exatecan

The effective payload in T-DXd, Dxd (derivative of Exatecan), is a highly cytotoxic compound obtained through multiple modifications. The development of this compound underwent a series of optimization stages: initially starting from camptothecin, then transitioning to Irinotecan (CPT-11), followed by SN-38, and ultimately evolving into Exatecan mesylate (DX-8951f) (Nakada et al., 2019; Xu et al., 2019). Notably, DX-8951f is a water-soluble CPT, and compared to other CPT derivatives, it exhibits superior TopoⅠ inhibitory activity and antitumor effects. Additionally, DX-8951f demonstrates significant efficacy against multi-drug resistant cells mediated by P-glycoprotein (P-gp) (De Jager et al., 2000). *In vitro* experimental data indicate that the cytotoxicity of DXd is approximately 10 times that of SN-38, suggesting its potent and broad-spectrum antitumor activity (Nakada et al., 2019). It’s worth noting that TopoⅠ inhibitors have been relatively less utilized in breast cancer treatment, providing a unique advantage for DXd: for breast cancer patients who have previously been treated with microtubule inhibitors, the use of a drug with this novel mechanism of action can circumvent resistance issues (Kumar and Sherman, 2023).

### 3.3 Payload of SG: SN-38, metabolite of irinotecan

Irinotecan is a derivative of camptothecin and has been extensively used in the treatment of solid tumors for over 20 years. Its active metabolite, SN-38, exhibits anticancer activity that is 100–1000 times greater than that of Irinotecan itself. Both of these compounds act as inhibitors of DNA topoisomerase I (TopoⅠ), allowing SN-38 to induce cancer cell apoptosis by interfering with DNA replication (Bailly, 2019; Goldenberg and Sharkey, 2020). Metabolically, SN-38 is primarily converted in the liver to its inactive form, SN-38G, via the uridine diphosphate-glucuronosyltransferase (UGT1A) enzyme and is subsequently excreted from the body. However, in the intestines, a portion of SN-38G is reactivated to SN-38 by bacterial β-glucuronidase, which is one of the primary reasons for the diarrhea induced by Irinotecan (Dong et al., 2019). Similar to Irinotecan, SN-38 has a range of adverse effects, including neutropenia, diarrhea, mucositis, liver damage, and more rarely, retinal disorders. Notably, neutropenia and diarrhea are the most common side effects (Bailly, 2019; Boileve et al., 2019). It's important to note that the risk of adverse reactions from SN-38 is directly proportional to its blood concentration. The glucuronidation of SN-38 is closely related to the activity of the UGT1A enzyme, and the UGT1A gene exhibits polymorphism with multiple isoforms. Studies have shown that enzymes with catalytic activities like UGT1A16/28, UGT1A73/3, and UGT1A9*1/*1 have lower activity, making them potential biomarkers for predicting severe adverse reactions from Irinotecan (Inoue et al., 2013). Additionally, certain drugs, such as Pazopanib and Erlotinib, can inhibit the catalytic activity of the UGT1A enzyme, leading to increased drug interactions and toxicity (Liu et al., 2010; Iwase et al., 2019).

## 4 Strategy of linker

In the design of Antibody-Drug Conjugates (ADCs), linkers play a pivotal role. They not only ensure the stable binding between the antibody and the active payload, preventing premature release of the payload, but also significantly influence the pharmacokinetics (PK), therapeutic index, and safety of the drug (Jain et al., 2015). Based on their structure and functionality, linkers can be categorized into cleavable and non-cleavable types. Cleavable linkers are sensitive to the intracellular environment and can be cleaved under specific conditions, such as the action of proteases or in low pH environments, leading to the release of cytotoxic molecules. In contrast, non-cleavable linkers require proteolytic degradation of the ADC’s antibody portion to release the active payload (von Arx et al., 2023).

T-DM1 is composed of the anti-HER2 antibody trastuzumab, a non-cleavable thioether linker N-maleimidomethyl cyclohexane-1-carboxylate (MCC), and a potent microtubule-depolymerizing maytansinoid derivative, DM1 (55). Its drug-to-antibody ratio (DAR) is 3.5 (56).

**FIGURE 1:**
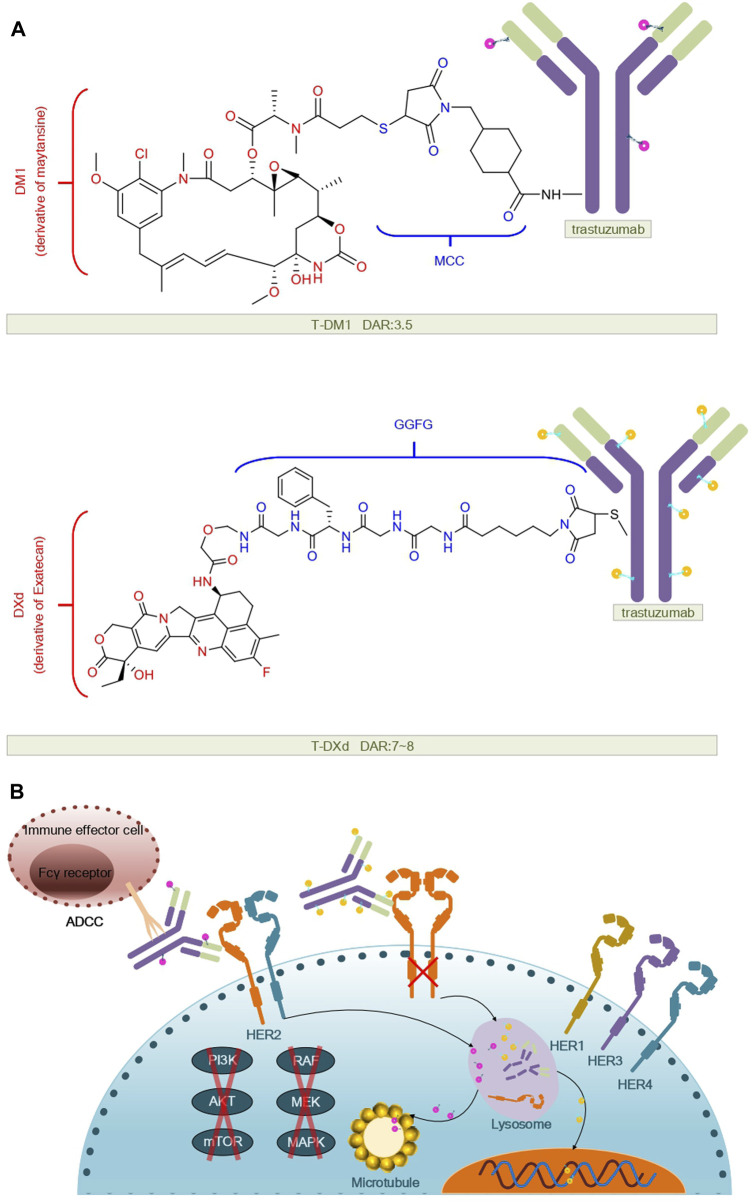
Structural and mechanistic sketch of T-DM1 and T-DXd. **(A)** Structure of T-DM1 and T-DXd. **(B)** Mechanism of action of T-DM1 and T-DXd.

T-Dxd is constructed by conjugating trastuzumab with a cleavable tetrapeptide linker maleimide glycine-glycine-phenylalanine-glycine (GGFG) and the Topo I inhibitor exatecan (Yu et al., 2022). The enhanced therapeutic efficacy of T-Dxd may be attributed to its higher drug-to-antibody ratio (DAR: 7–8), the unique cleavable GGFG peptide linker, and the superior membrane permeability of the Dxd payload. These attributes confer DS-8201a with a stronger bystander effect against non-targeted cancer cells (Xu et al., 2019). The structure and mechanism regarding T-DM1, T-DXd are summarized in Figure 1.

**FIGURE 2:**
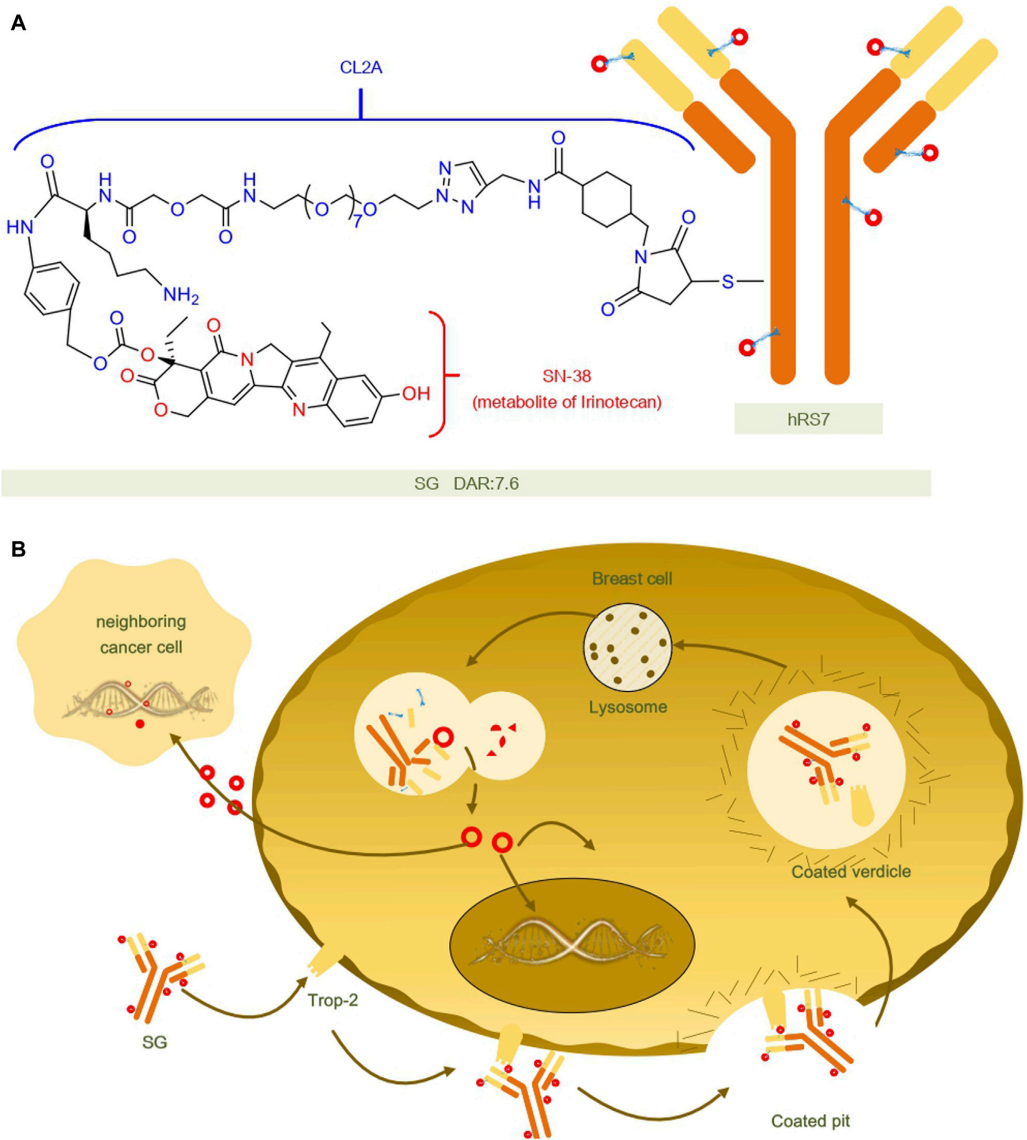
Structural and mechanistic sketch of SG. **(A)** Structure of SG. **(B)** Mechanism of action of SG.

Sacituzumab govitecan (SG) is composed of a humanized anti-trophoblast cell-surface antigen 2 (Trop-2) monoclonal antibody (hRS7), a cleavable CL2A linker, and the DNA topoisomerase I inhibitor SN-38.Upon entry into the body and binding to Trop-2 expressing tumor cells, hRS7 is internalized and the covalent bond between the junction and SN-38 is cleaved and the active form of SN-38 is released at low pH (e.g., in lysosomes), or in the tumor microenvironment, or by enzymatic degradation (Starodub et al., 2015; Syed, 2020).Additionally, SG exhibits a bystander effect where SN-38 can extend beyond targeted Trop-2 cancer cells and exert a cytotoxic impact on adjacent cells, regardless of their Trop-2 expression status (Kaboli et al., 2022). The structure and mechanisms regarding SG are summarized in Figure 2.

## 5 Completed clinical trials

### 5.1 Results of completed clinical trials of T-DM1 for breast cancer treatment

In the treatment of HER2-positive advanced breast cancer: The EMILIA clinical trial (NCT00829166) compared the efficacy of T-DM1 with lapatinib plus capecitabine in patients with HER2-positive advanced breast cancer who had previously received trastuzumab and taxanes (Verma et al., 2012). The results of the study revealed that the median progression-free survival (mPFS), median overall survival (mOS), and objective response rate (ORR) of the T-DM1 group were significantly better than those of the lapatinib plus capecitabine group, which were 9.6 months: 6.4 months; 30.9 months: 25.1 months; and 43.6%: 30.8%, respectively. In the safety assessment, the incidence of grade 3 or 4 adverse events related to T-DM1 was 41%, lower than the 57% observed in the lapatinib plus capecitabine group. Although the T-DM1 group had a higher incidence of thrombocytopenia and elevated serum transaminase levels, the lapatinib combined with capecitabine group had a more pronounced incidence of diarrhea, nausea, vomiting, and hand-foot syndrome (Diéras et al., 2017).Another clinical trial named TH3RESA (NCT01419197) aimed to evaluate the efficacy and safety of T-DM1 compared to physician’s choice of treatment in HER2-positive metastatic breast cancer patients who had previously received trastuzumab, lapatinib, and taxanes. The results demonstrated that the T-DM1 treatment group had a significantly longer mOS than the physician’s choice treatment group at 22.7 months:15.8 months. In terms of safety, T-DM1 exhibited good tolerability, especially when compared to the trastuzumab-based physician’s choice treatment regimen, even though the treatment duration for T-DM1 was nearly double that of the latter. In conclusion, the TH3RESA trial further confirmed the significant efficacy and favorable tolerability of T-DM1 in the treatment of HER2-positive advanced breast cancer, especially for patients who are refractory to other HER2-targeted therapies (Krop et al., 2014; Krop et al., 2017).

In the treatment of early-stage HER2-positive breast cancer: The ATEMPT trial focused on a comparative analysis of the safety of T-DM1 *versus* paclitaxel plus trastuzumab (TH) in patients with early stage HER2-positive breast cancer. In terms of quality of life, based on FACT-B measurements, the quality of life was notably lower in the TH treatment group. Regarding clinically relevant toxicities, the incidence of toxicity was similar between the T-DM1 and TH treatment groups. However, the hair loss rate was significantly lower in the T-DM1 group (0%) compared to the TH group (41%) (Tolaney et al., 2021). In the third phase of the KAITLIN study (NCT01966471), researchers explored the potential of improving the treatment efficacy and reducing toxicity in HER2-positive early breast cancer (EBC) by using trastuzumab emtansine (T-DM1) as a replacement for taxanes and trastuzumab. After a median follow-up of 57.1 months and 57.0 months, both treatment groups, AC-THP and AC-KP, demonstrated similar outcomes in terms of invasive disease-free survival (IDFS), with 3-year IDFS rates of 94.2% and 93.1%, respectively, in the overall population. The proportion of patients who completed all 18 treatment cycles was 88.4% in the AC-THP group and 65.0% in the AC-KP group, with the primary reason for discontinuation being laboratory abnormalities induced by T-DM1 (12.5%). The two groups exhibited similar rates of grade ≥3 and severe adverse events, at 55.4% *versus* 51.8% and 23.3% *versus* 21.4%, respectively. It is noteworthy that the KP group showed a clinically meaningful reduction in global health status deterioration compared to the THP group (hazard ratio [HR] 0.71; 95% confidence interval, 0.62–0.80). In summary, although the primary endpoints were not met as expected, both treatment strategies demonstrated favorable IDFS outcomes. Therefore, for high-risk HER2-positive EBC patients, the standard of care remains to be Trastuzumab plus Pertuzumab in combination with chemotherapy (Krop et al., 2022).

T-DM1 is used in the adjuvant or neoadjuvant aspects of breast cancer: The KATHERINE trial (NCT01772472) demonstrated that adjuvant therapy with T-DM1 significantly reduces the risk of recurrence or death in patients with early-stage HER2-positive breast cancer. This trial included 1486 patients who still had residual invasive disease after receiving neoadjuvant chemotherapy and HER2-targeted therapy. They were randomly assigned to receive 14 cycles of either T-DM1 or trastuzumab as adjuvant treatment. The results revealed that the invasive disease-free survival rate was significantly higher in the T-DM1 group compared to the trastuzumab group. Specifically, 12.2% of patients in the T-DM1 group experienced invasive disease or death, compared to 22.2% in the trastuzumab group. The 3-year invasive disease-free survival rate for T-DM1 was 88.3%, while it was 77.0% for trastuzumab. These findings further underscore the advantage of T-DM1 in reducing the risk of invasive disease or death, with a risk reduction of 50% (von Minckwitz et al., 2019). For patients in stages II to III, the KRISTINE trial (NCT02131064) evaluated the efficacy and safety of T-DM1 combined with pertuzumab (T-DM1+P) as a neoadjuvant treatment compared to the conventional regimen of docetaxel plus carboplatin with dual HER2-targeted blockade. In this trial, patients received treatment every 3 weeks for a total of 18 cycles, encompassing both neoadjuvant and adjuvant treatment phases. The primary endpoint for neoadjuvant treatment was the pathological complete response rate (pCR). The results revealed that the pCR rate for the T-DM1+P regimen was 44.4%, while the traditional regimen of docetaxel + carboplatin with dual HER2 blockade achieved a pCR rate of 55.7%. Notably, during the neoadjuvant treatment phase, the rate of grade 3 or higher adverse events (AEs) for the T-DM1 regimen was only 13.0%, significantly lower than the 64.4% observed in the conventional regimen. Three-year follow-up efficacy data indicated a higher risk of event-free survival (EFS) for the T-DM1+P regimen, with a hazard ratio (HR) of 2.61 (95% CI, 1.36–4.98), and more local progression events before surgery. However, post-surgery, both treatment regimens exhibited comparable risks for invasive disease-free survival (IDFS), with an HR of 1.11 (95% CI, 0.52–2.40). This suggests that both regimens had similar efficacy in the adjuvant treatment phase. In summary, while the traditional docetaxel + carboplatin regimen demonstrated a higher pCR rate in the neoadjuvant treatment phase, the T-DM1 regimen showed comparable long-term efficacy and superior safety in the adjuvant treatment phase (Hurvitz et al., 2018; Hurvitz et al., 2019; de Haas et al., 2023). WSG-ADAPT-TP trail demonstrated that downscaled neoadjuvant therapy with T-DM1, T-DM1 + ET, and trastuzumab + ET was comparable for HER2 + EBC, and that omission of adjuvant chemotherapy in pCR patients did not impair iDFS(71).

KAMILLA is a phase IIIb study specifically targeting patients with HER2-positive breast cancer, with a particular focus on the efficacy of T-DM1 in patients with brain metastases. In this study, 2002 global patients (Group 1) and 181 Asian patients (Group 2) were enrolled. Notably, approximately 70% of these patients had already undergone at least two prior treatment lines in the context of metastatic disease. In terms of safety, the overall adverse event rate for Group 2 was 51.4%, primarily attributed to grade ≥3 thrombocytopenia. However, this was not associated with grade ≥3 bleeding events, and most of these patients (128/138) fully recovered. In contrast, the overall adverse event rate for Group 1 was 23.1%. Regarding efficacy, the median progression-free survival for Group 1 and Group 2 was 6.8 months and 5.7 months, respectively, while their median overall survival was 27.2 months and 29.5 months, respectively (Wuerstlein et al., 2022). Additionally, 398 patients had brain metastases at the start of the study. Among the 126 patients with measurable brain metastases, the best overall response rate was 21.4%, and the clinical benefit rate was 42.9%. For these 398 patients with brain metastases, their median progression-free survival and overall survival were 5.5 months and 18.9 months, respectively (Montemurro et al., 2020). In summary, T-DM1 demonstrated favorable safety and efficacy profiles both globally and specifically within the Asian patient population, especially notable in patients with brain metastases.

The MARIANNE trial (NCT01120184) was designed to compare the efficacy and safety of the trastuzumab plus paclitaxel arm, the T-DM1 arm, and the T-DM1 plus pertuzumab arm as first-line treatment for HER2-positive progressive or recurrent locally advanced or metastatic breast cancer. The study results revealed that the progression-free survival (PFS) for these three treatment regimens was 13.7 months, 14.1 months, and 15.2 months, respectively. However, neither of the two T-DM1-based experimental groups achieved a PFS superior to the group treated with pertuzumab plus paclitaxel. In terms of response rates, the pertuzumab plus paclitaxel group had a rate of 67.9%, the T-DM1 group had a rate of 59.7%, and the T-DM1 plus pertuzumab group had a rate of 64.2%. The corresponding durations of response were 12.5 months, 20.7 months, and 21.2 months, respectively. Regarding the incidence of grade ≥3 adverse events, the pertuzumab plus paclitaxel group (54.1%) showed a slightly higher rate compared to the T-DM1 group (45.4%) and the T-DM1 plus pertuzumab group (46.2%) (Perez et al., 2017).

### 5.2 Results of completed clinical trials of T-DXd for breast cancer treatment

In the treatment research of HER2-positive metastatic breast cancer, the DESTINY-Breast01 trial conducted an in-depth evaluation of T-DXd treatment for adult patients who had previously received T-DM1 therapy. The study first established the recommended dosage and subsequently assessed its efficacy and safety. Among the 184 patients treated with the recommended dose (5.4 mg/kg), 60.9% responded to the treatment, with a median response duration of 14.8 months and a progression-free survival of 16.4 months. Adverse reactions during the treatment included a decrease in neutrophil count (20.7%), anemia (8.7%), nausea (7.6%), and interstitial lung disease (ILD) (13.6%). The study concluded that T-DXd treatment exhibits significant antitumor activity, but attention should be given to specific adverse reactions (Modi et al., 2020a). In the DESTINY-Breast02 trial, 608 unresectable or HER2-positive metastatic breast cancer patients were randomly assigned to receive T-DXd (5.4 mg/kg, every 3 weeks) or treatment chosen by their physician. The results showed that the median progression-free survival for the T-DXd treatment group was 17.8 months, compared to 6.9 months for the control group (HR 0.36 [0.28–0.45]; *p* < 0.0001). Common adverse reactions in the T-DXd group included nausea, vomiting, hair loss, fatigue, diarrhea, and palmar-plantar erythrodysesthesia, with a 10% incidence rate of interstitial lung disease related to T-DXd. The study confirmed that T-DXd treatment offers significant therapeutic benefits and a favorable benefit-risk profile for HER2-positive metastatic breast cancer patients (André et al., 2023). The DESTINY-Breast03 trial compared the efficacy and safety of T-DXd and T-DM1 in HER2-positive metastatic breast cancer patients who had previously received trastuzumab and corticosteroids. The findings revealed that the median progression-free survival for the T-DXd group was 28.8 months, while it was 6.8 months for the T-DM1 group. In terms of the 24-month overall survival rate, the T-DXd group achieved 77.4%, compared to 69.9% for the T-DM1 group (HR 0.55). Additionally, the overall response rate was 79.7% for the T-DXd group and 34.2% for the T-DM1 group. Both treatment regimens had similar rates of adverse events, but the incidence of interstitial lung disease or pneumonia related to T-DXd was higher at 15%. However, compared to T-DM1, T-DXd treatment maintained patients’ health-related quality of life (QoL) and delayed the deterioration of QoL (77–79). In summary, the research data supports the significant therapeutic benefits of T-DXd treatment for HER2-positive metastatic breast cancer patients, demonstrating its outstanding progression-free survival, overall survival, and manageable toxicity.

In patients with HER2-low expressing metastatic breast cancer, the DESTINY-Breast04 study evaluated the efficacy and safety of T-DXd compared to the standard chemotherapy chosen by physicians. Among the 557 participants, 88.7% were identified as hormone receptor-positive. The results indicated that the median progression-free survival for the HER2-low expressing metastatic breast cancer cohort treated with T-DXd was 10.1 months, compared to 5.4 months for those receiving standard treatment (hazard ratio of 0.51, *p* < 0.001). Furthermore, the overall survival was notably longer in the T-DXd treatment group, reaching 23.9 months, in contrast to 17.5 months in the control group (hazard ratio of 0.64, *p* = 0.003). In the hormone receptor-negative subset, the median progression-free survival for T-DXd was 8.5 months (95% CI, 4.311.7), while the treatment chosen by physicians resulted in 2.9 months (95% CI, 1.45.1) (hazard ratio of 0.46; 95% CI, 0.24–0.89). The median overall survival for this subset was 18.2 months (95% CI, 13.6 to not estimable) and 8.3 months (95% CI, 5.6–20.6) respectively (hazard ratio of 0.48; 95% CI, 0.24–0.95). However, the study also revealed that 52.6% of patients treated with T-DXd experienced adverse reactions of grade 3 or higher, with 12.1% manifesting drug-related interstitial lung disease. Despite these adverse events, the trial concluded that, compared to standard chemotherapy, T-DXd significantly prolongs the progression-free and overall survival of patients with HER2-low expressing metastatic breast cancer, highlighting its potential as a targeted treatment option for this patient population (Modi et al., 2022b).

The primary objective of the DAISY trial was to evaluate the efficacy of T-DXd in patients with metastatic breast cancer with HER2 overexpression, HER2 low expression, and HER2 non-expression. Among the 177 participants, they were categorized into three groups based on their HER2 expression status. The primary endpoint of the study was the confirmed objective response rate (ORR). The results indicated that the ORR for the first group was 70.6% (95% CI 58.3–81), for the second group was 37.5% (95% CI 26.4–49.7), and for the third group was 29.7% (95% CI 15.9–47) (Mosele et al., 2023).

The TUXEDO-1 trial aimed to explore the therapeutic effects of T-DXd on HER2-positive breast cancer patients with active brain metastases. This study involved 15 patients who were newly diagnosed or had progressive brain metastases. In the intention-to-treat (ITT) population, the response rate reached 73.3%, and the median progression-free survival (PFS) was 14 months. At the median follow-up of 12 months, the overall survival was not yet determined, suggesting that intracranial activity has clinical relevance. All adverse events were graded according to the Common Terminology Criteria for Adverse Events (CTCAE) v.5.0 of the National Cancer Institute. Among them, 4 patients experienced a total of 6 severe adverse events, including 2 cases of interstitial lung disease (Bartsch et al., 2022).

The objective of the NCT02564900 trial was to assess the safety and tolerability of DS-8201a in patients with advanced solid malignancies. During the dose-expansion phase, patients were primarily divided into five different cohorts, including those with HER-2 positive or HER-2 low-expressing advanced breast cancer, where HER-2 positive patients had previously received standard T-DM1 treatment. The study results showed that the ORR for HER-2 positive patients was 59.5%, while for HER-2 low-expression it was 37.0% (Tamura et al., 2019; Modi et al., 2020b).

### 5.3 Results of completed clinical trials of SG for breast cancer treatment

The IMMU-132–01 trial, an open, single-arm, multicenter phase I/II trial, is comprised of a dose-escalation and dose-expansion phase. This trial primarily aims to assess the safety and efficacy of sacituzumab govitecan (SG) in the treatment of patients with advanced epithelial cancer. Notably, TROP-2 is highly expressed in the tissues of patients with various epithelial cancers. During the dose-expansion phase, the trial enrolled 495 patients, irrespective of their TROP-2 expression levels. Among these, 108 were patients with triple-negative breast cancer (TNBC) who had received at least two prior anticancer treatments for metastatic TNBC, and 54 patients had HR+/HER2-metastatic breast cancer, having received a minimum of two prior metastatic breast cancer treatments. SG was administered intravenously at a dosage of 10 mg/kg on days 1 and 8 of the 21-day cycle until disease progression or unacceptable toxicity was observed.

The triple-negative breast cancer cohort yielded an objective response rate (ORR) of 33.3%, median duration of remission (mDOR) of 7.7 months, clinical benefit rate (CBR) of 45.4%, median progression-free survival (mPFS) of 5.5 months, and overall survival (OS) of 13.0 months (Bardia et al., 2019).

In the cohort of patients with HR+/HER2-metastatic breast cancer, following a median follow-up period of 11.5 months, the objective response rate (ORR) was 31.5%, median duration of remission (DOR) stood at 8.7 months, median progression-free survival (mPFS) was 5.5 months, and the median overall survival (mOS) was recorded as 12 months (Kalinsky et al., 2020; Bardia et al., 2021a).

SG exhibited promising activity in treating HR+/HER2-metastatic breast cancer. Currently, a further randomized phase III trial (TROPiCS-02,NCT03901339) is underway (Kalinsky et al., 2020). This trial’s primary objective is to evaluate and compare the efficacy and safety of SG with the treatment of choice (TPC) in participants with HR+/HER2-metastatic breast cancer. HR+/HER2- MBC patients are often administered endocrine therapy in combination with targeted agents such as CDK 4/6 inhibitors, as well as PI3K and mTOR inhibitors, which usually serve as the initial and second or third line treatment options. Following the tolerance of endocrine therapy, the response rate to later line therapy also diminishes. The results of the TROPiCS-02 study will offer reliable efficacy and safety data for SG, potentially paving the way for a novel and effective postline treatment option for patients with HR+/HER2-metastatic breast cancer (Rugo et al., 2020).

The ASCENT trial is an international, multicenter, open-label, randomized phase III trial aimed at comparing the efficacy of sacituzumab govitecan (SG) with the treatment of physician’s choice (TPC), which includes eribulin, vincristine, capecitabine, or gemcitabine, in patients with recurrent or refractory metastatic triple-negative breast cancer (TNBC). The trial involved a total of 529 TNBC patients, including 61 with brain metastases and 468 without, at baseline. These patients were randomly assigned to either the SG group (235 patients) or the TPC group (233 patients). The SG group exhibited a median progression-free survival of 5.6 months, compared to 1.7 months in the TPC group, and a median overall survival of 12.1 months *versus* 6.7 months in the TPC group (Bardia et al., 2021b).

For biomarker analysis, biopsy or surgical specimens were collected at the onset of the study. Validated immunohistochemical assays and histochemical scoring were used to determine TROP-2 expression levels, while patients’ BRCA1/2 gene mutation statuses were gathered at baseline for subsequent subgroup analyses. Out of 468 evaluable patients, 290 had available Trop-2 expression data (152 (64%) in the SG group and 139 (60%) in the TPC group). Additionally, 292 patients had known BRCA1/2 gene mutations (149 (63%) in the SG group and 143 (61%) in the TPC group). In patients with high, medium, and low TROP-2 expression levels, mPFS and mOS for SG *versus* TPC groups were, respectively, 6.9 months vs. 2.5 months, 5.6 months vs. 2.2 months, and 2.7 months vs. 1.6 months; while mOS was 14.2 months vs. 6.9 months, 14.9 months vs. 6.9 months, and 9.3 months vs. 7.6 months. ORRs were also higher in the SG group (44%, 38%, 22%) compared to the TPC group (1%, 11%, 6%). Similar trends were observed in the SG group among patients with or without BRCA1/2 gene mutations (Bardia et al., 2021c).

In terms of pre-enrollment diagnosis, the study included 146 patients who did not have TNBC at the initial diagnosis (70 (30%) in the SG group and 76 (33%) in the TPC group). In a subgroup analysis comparing the clinical benefit between the SG and TPC groups, the mPFS was 4.6 months vs. 2.3 months, and the mOS was 12.4 months for both groups. The efficacy of SG was similar in patients with TNBC at the time of initial diagnosis and in patients without TNBC at the time of initial diagnosis (O Shaughnessy et al., 2022; O Shaughnessy et al., 2021).

Regarding treatment duration, 33/235 and 32/233 patients (both 14%) in the SG and TPC groups, respectively, had only received one treatment in the metastatic setting and relapsed after 12 months of (neo) adjuvant chemotherapy. SG extended both PFS (5.7 months vs. 1.5 months) and OS (10.9 months vs. 4.9 months) in this subgroup, with a consistent overall safety profile compared to the overall population. Thus, SG improved survival in metastatic TNBC in this second-line setting, as well as in postline therapy (Carey et al., 2022).

The FDA approved T-DM1 in 2013 for the treatment of patients with HER2-positive metastatic breast cancer (MBC) who had previously been treated with trastuzumab and a taxane. This approval was based on the results of the EMILIA trial. In 2019, the FDA expanded the indications for T-DM1 to include adjuvant treatment for early HER2-positive breast cancer patients who had residual invasive disease in the breast or lymph nodes after neoadjuvant treatment with a taxane and trastuzumab-based regimen, as investigated in the KATHERINE trial. T-DXd was approved in December 2019 for the treatment of patients with HER2-positive unresectable or metastatic breast cancer who had received at least two prior anti-HER2-directed treatments. This approval was based on the results of the DESTINY-Breast01 trial. Additionally, in August 2022, T-DXd was approved for the treatment of patients with unresectable or metastatic HER2-low breast cancer. Inspired by the results of the IMMU-132-01 and ASCENT trials, the FDA granted accelerated approval to sacituzumab govitecan in April 2020 for the treatment of adult patients with metastatic triple-negative breast cancer (mTNBC) who had received at least two prior therapies for metastatic disease (Syed, 2020). (All clinically relevant data are organized in Table 1).

**TABLE 1:** Completed clinical trials.

NCT Number/Phase	Type of disease	Prior therapy	Interventions	Number of cases	Efficacy data	grade≥3 AE
T-DM1						
EMILIA NCT00829166/Ⅲ (Diéras et al. 2017)	HER2+ Locally A/MBC	Trastuzumab/Paclitaxel	E: T-DM1	495	mPFS: 9.6 months mOS: 30.9 months	40.8%
			C: Lapatinib + Capecitabine	496	mPFS: 6.4 months mOS: 25.1 months	57.0%
TH3RESA NCT01419197/Ⅲ (Krop et al., 2014; Krop et al., 2017)	HER2+ MBC	≥2 HER2-targeted therapy	E: T-DM1	404	mPFS: 6.2 months mOS: 22.7 months	40%
			C:TPC	198	mPFS: 3.3 months mOS: 15.8 months	47%
ATEMPT NCT01853748/Ⅱ (Tolaney et al., 2021)	HER2+ EBC	—	E:T-DM1	383	3 years IDFS:97.8%	CRT:46%
			C:TH	114	3 years IDFS:93.4%	CRT:47%
KAITLIN NCT01966471/Ⅲ (Krop et al., 2022)	Operable HER2+ EBC	—	E: AC-KP	832	3 years IDFS:94.2%	51.8%
			C: AC-THP	918	3 years IDFS:93.1%	55.4%
KATHERINE NCT01772472/Ⅲ (von Minckwitz et al., 2019)	HER2+ PBC (adjuvant therapy)	Surgical	E:T-DM1	743	3 years IDFS: 88.3%	25.7%
			C: Trastuzumab	743	3 years IDFS:77.0%	15.4%
KRISTINE NCT02131064/Ⅲ (Hurvitz et al., 2018; Hurvitz et al., 2019; de Haas et al., 2023)	HER2+ BC (neoadjuvant therapy)	—	E:T-DM1+ pertuzumab	223	pCR: 44·4%	13%
					3 years IDFS: 93%	
					3 years EFS: 85%	
			C:docetaxel, carboplatin and trastuzumab + pertuzumab	221	pCR:55·7%	64%
					3 years IDFS: 92%	
					3 years EFS: 94%	
WSG-ADAPTTP NCT01779206/Ⅱ/Ⅲ (Harbeck et al., 2023)	HR+/HER2+ EBC	—	T-DM1	125	IDFS: 88.9%	—
			T-DM1+ET	125	IDFS: 85.3%	—
			Trastuzumab + ET	125	IDFS: 84.6%	—
KAMILLA NCT01702571/Ⅲ (Montemurro et al., 2020; Wuerstlein et al., 2022)	HER2+ Locally a/MBC	Anti-HER2 and Chemotherapy	E = T-DM1	2002(All participant)	mPFS:6.8 months OS:27.2 months	—
			E = T-DM1	181(Asian participant)	mPFS:5.7 months OS:29.5 months	—
MARIANNE NCT01120184/Ⅲ (Perez et al., 2017)	HER2+ Progressive or Recurrent Locally a/MBC		E = T-DM1 + pertuzumab	365	PFS:15.2 months	54.1%
			E = T-DM1	363	PFS:14.1 months	46.2%
			E = trastuzumab + taxane	367	PFS:13.7 months	45.4%
T-DXd						
DESTINY-Breast01 NCT03248492/Ⅱ (Modi et al., 2020a)	HER2+ Unresectable and/or MBC	T-DM1	E = T-DXd	184	PFS:16.4 months	—
DESTINY-Breast02 NCT03523585/Ⅲ (André et al., 2023)	HER2+ Unresectable and/or MBC	T-DM1	C = Anthracycline + T-DM1+Pertuzumab + Taxane	928	PFS:57.0 months	51.8%
			C:capecitabine + trastuzumab/lapatinib	202	PFS:6.9 months	44%
DESTINY-Breast03 NCT03529110/Ⅲ (Cortes et al., 2022; Curigliano et al., 2023; Hurvitz et al., 2023)	HER2+ Unresectable and/or MBC	Trastuzumab/Paclitaxel	E:T-DXd	261	mPFS: 28·8 months 24 months OS rate: 77.4%	45.1%
			C:T-DM1	263	mPFS: 6·8 months 24 months OS rate: 69.9%	39.8%
DESTINY-Breast04 NCT03734029/Ⅲ (Modi et al., 2022b)	HER2 low, Unresectable and/or MBC	chemotherapy	E:T-DXd	373	mPFS/mOS: 9.9/23.4 months	52.6%
				HER2+:331	HER2+mPFS/mOS: 10.1/23.9 months	
				HER2 low:42	HER2 low mPFS/mOS: 8.5/18.2 months	
			C:TPC	184	mPFS/mOS: 5.1/16.8 months	67.4%
				HER2+:163	HER2+mPFS/mOS: 5.4/17.5 months	
				HER2 low:21	HER2- mPFS/mOS: 2.9/8.3 months	
DAISY NCT04132960/Ⅱ (Mosele et al., 2023)	ABC	—	T-DXd	HER2 overexpressing; HER2 IHC 3+ or ERBB2 ISH + *n* = 86>	ORR: 70.6% (95% CI,58.3–81)	—
				HER2 low; HER2 IHC 2+ or ERBB2 ISH– or IHC 1 + *n* = 49	ORR: 37.5% (95% CI,26.4–49.7)	
				HER2 non-expressing; HER2 IHC 0 *n* = 51	ORR:29.7% (95% CI,15.9–47)	
TUXEDO-1 NCT04752059/Ⅱ (Bartsch et al., 2022)	HER2+ BC and brain metastases	—	T-DXd	15	ORR: 73.3%	53.3%
NCT02564900/Ⅰ (Tamura et al., 2019; Modi et al., 2020b)	Advanced Solid Malignant Tumors	—	T-DXd	HER2+: 115	HER2+ ORR: 59·5% (95% CI 49·7–68·7)	—
				HER2 low: 54	HER2 low ORR:37.0% (95% CI, 24.3%–51.3%)	
SG						
IMMU-132–01 NCT01631552 Ⅰ/Ⅱ	TNBC(7)	—	E: SG	108	mPFS:5.5 months	Neutropenia
					OS: 13.0 months	
	HR+/HER2- MBC(85, 86)	—	E: SG	54	mPFS:5.5 months	Neutropenia:50.0%
					mOS:12.0 months	Constipation:7.4%
ASCENT NCT02574455 Ⅲ (Bardia et al., 2021b)	MTNBC	—	E:SG	235	mPFS:5.7 months	Neutropenia:51.0%
					mOS:12.1 months	
			C:TPC	233	mPFS:1.7 months	Neutropenia:33.0%
					mOS:6.7 months	

Note: E is the experimental group, C is the control group, n is the number of cases, and AE, is the adverse event. HER2+:HER2-Positive; HER2-:HER2-negative; ABC: advanced metastatic breast cancer; MBC:metastatic breast cancer; PBC:Primary Breast Cancer; ET: endocrine therapy; TPC: Treatment of physician’s choice; A: Doxorubicin C:cyclophosphamide T:Paclitaxel H: Trastuzumab P: pertuzumab; CRT: clinically relevant toxicities.

## 6 Ongoing clinical trials

Ongoing clinical trials of T-DM1, T-DXd, and SG for the treatment of breast cancer were searched through ClinicalTrials.gov as of 15 October2023, to explore the research progress of the three drugs, T-DM1, T-DXd, and SG, in the treatment of breast cancer. Clinical trials with suspended, terminated, orphaned, and unknown status were excluded, and all clinical trials of T-DXd, SG for the treatment of breast cancer were included, and for T-DM1, Phase I and II clinical trials for the treatment of breast cancer with first published results and some with enrollment data after 2020, as well as all Phase III clinical trials, were included.

### 6.1 Monotherapy

Monotherapy is designed to further investigate the use of T-DM1, T-DXd, and SG in addition to the approved indications. The primary focus of these studies will be to compare the advantages and disadvantages of T-DM1, T-DXd, and SG with conventional drug regimens (Relevant clinical trials are organized in Table 2).

**TABLE 2:** Clinical trials of monotherapy.

Conditions	NCT Number/Phase/Situation of patients	Interventions	Enrollment reported in available data	Primary completion
T-DM1				
HER2+	NCT03084939/Ⅲ	E = T-DM1	—	23 November 2018
	Chinese Patients With HER2-Positive Locally A/MBC Who Have Received Prior Trastuzumab-Based Therapy	C = lapatinib + capecitabine		
	NCT04924699/Ⅲ	E = MRG002	—	September 2023
	Unresectable Locally A/MBC	C = T-DM1		
HER2-	NCT01975142/Ⅱ (Jacot et al., 2019)	E = T-DM1	n = 154	13 February 2018
	MBC, HER2 Negative Primary Tumor		mPFS = 4.8 months	
			mOS = 9.5 months	
T-DXd				
HER2+	NCT04622319/Ⅲ DESTINY-Breast05	E = T-DXd	—	31 October 2025
	Participants With High-Risk HER2-Positive PBC Who Have Residual Invasive Disease in Breast or Axillary Lymph Nodes Following Neoadjuvant Therapy	C = T-DM1		
	NCT04739761/Ⅲ(DESTINY-Breast12)	E = T-DXd	—	9 February 2024
	Patients With or Without Baseline Brain Metastasis With Previously Treated Advanced/Metastatic HER2-Positive Breast Cancer			
	NCT05113251/Ⅲ(DESTINY-Breast11)	E A = T-DXd	—	25 December 2023
	High-risk HER2-positive Early-stage Breast Cancer	E B = T-DXd followed by THP C = doxorubicin and cyclophosphamide, followed by THP		
	NCT05744375/Ⅰ/Ⅱ(TRANSCENDER Study) in first-line Treatment of HER2-positive Locally advanced or MBC, Patients Considered Resistant to Trastuzumab + Pertuzumab + Taxane Due to Early Relapse	E = T-DXd	—	1 July 2027
	NCT04752059/Ⅰ/Ⅱ	E = T-DXd	—	1 May 2023
	HER2-positive Breast Cancer Patients With Newly Diagnosed or Progressing Brain Metastases			
	NCT05704829/Ⅰ/ⅡNeoAdjuvant HER2+ Early Breast Cancer	low-intermediate risk for recurrence	—	June 2026
		E = T-DXd		
		C=Chemotherapy + T + P intermediate-high risk for recurrence		
		E = T-DXd		
		C=Chemotherapy + T + P		
HER2 Low	NCT05953168/Ⅰ、Ⅱ	E = T-DXd	—	August 2024
	First-line Treatment Locally A/MTNBC, Luminal Androgen Receptor Subtype With Low HER2 Expression			
	NCT04494425/Ⅲ(DESTINY-Breast06)	E = T-DXd	—	29 December 2023
	HER2-Low, HR + BC Patients Whose Disease Has Progressed on Endocrine Therapy in the Metastatic Setting	C=Capecitabine/Paclitaxel/Nab-Paclitaxel		
E 1: HR-, HER2 low	NCT05950945/Ⅲ(DESTINY-Breast15) Unresectable and/or Metastatic HER2-low or HER2 Immunohistochemistry (IHC) 0 Breast Cancer	E:T-DXd	—	October 2027
E 2: HR-, HER2 IHC 0				
E 3: HR+, HER2-low				
E 4: HR+, HER2 IHC 0				
SG				
HER2-	NCT04595565/Ⅲ (Marmé et al., 2022)	E = SG	n_E_ = 45; n_c_ = 43	1 December 2026
	Primary HER2-BC	C= Capecitabine; Carboplatin; Cisplatin	AE (3–4):E = 66.7%; C = 20.9%	
	NCT04647916/Ⅱ	E = SG	—	17 February 2025
	HER2-BC and Brain Metastases			
HR+/HER2- MBC	NCT03901339/Ⅲ (Rugo et al., 2022a)	E = SG C=Eribulin; Capecitabine; Gemcitabine; Vinorelbine	n_E_ = 45; n_c_ = 43	October 2024
			mPF:E = 5.5; C = 4.0	
			NeutropeniaAE (3):E = 51%; C = 38%	
			DiarrheaAE (3): E = 9%; C = 1%	
	NCT04639986/Ⅲ	E = SG	—	31 March 2023
	Asian	C= Eribulin Mesylate Injection; Capecitabine Oral Product; Gemcitabine Injection; Vinorelbine injection		
	NCT05840211/Ⅲ	E = SG	—	September 2025
	Have Received Endocrine Therapy	C = doxorubicin and cyclophosphamide, followed by THP		
TNBC	NCT05101096/Ⅰ/Ⅱ	E = SG	—	March 2023
	Japanese With Advanced Solid Tumors or TNBC			
	NCT05382299/Ⅲ	E = SG	—	May 2027
	Previously Untreated Metastatic TNBC	C=Paclitaxel; nab-Paclitaxel; Gemcitabine; Carboplatin		
	NCT04454437/Ⅱ (Xu et al., 2023)	E = SG	n = 80	6 August 2021
	Chinese With Metastatic TNBC of at Least 2 Prior Treatments		mPFS = 5.55	
			AE (≥3) = 71.3%	

Notes: E is the experimental group, C is the control group, n is the number of cases, and AE, is the adverse event. HR-:HR-negative; HR+: HR-positive.

#### 6.1.1 T-DM1

NCT0308493 will evaluate the efficacy of T-DM1 *versus* chemotherapy in Chinese patients with HER2-positive locally advanced or metastatic breast cancer who have been previously treated with trastuzumab. NCT01975142 In a prospective phase II clinical trial, researchers evaluated the efficacy of trastuzumab-emtansine (T-DM1) in HER2-negative metastatic efficacy in patients with breast cancer (MBC) whose circulating tumor cells (CTCs) were HER2-positive. By screening, a limited number of HER2-negative MBC patients were found to have HER2-amplified CTCs. Of the 11 patients treated with T-DM1, only one patient achieved a partial response (Jacot et al., 2019).

#### 6.1.2 T-DXd

For patients with HER2-positive breast cancer, there are several relevant studies. Among them, DESTINY-Breast05 (NCT04622319) focuses on high-risk HER2-positive primary breast cancer patients who still have invasive lesions in the breast or axillary lymph nodes after receiving neoadjuvant therapy. Meanwhile, DESTINY-Breast11 (NCT05113251), in the neoadjuvant setting, compares the efficacy of T-DXd monotherapy with the subsequent use of THP and ddAC-THP in high-risk HER2-positive early-stage breast cancer patients. ADAPTHER2-IV (NCT05704829) investigates the application of T-DXd neoadjuvant therapy in HER2-positive early-stage breast cancer with medium-low/medium-high recurrence risk. DESTINY-Breast12 (NCT04739761) specifically targets late-stage/metastatic HER2-positive breast cancer patients who have previously received treatment and may or may not have baseline brain metastases. Additionally, the TRANSCENDER Study (NCT05744375) positions T-DXd as the first-line treatment for HER2-positive locally advanced or metastatic breast cancer (MBC) patients, who are considered resistant to trastuzumab + pertuzumab + taxanes due to early relapse. Lastly, NCT04752059 conducts research on HER2-positive breast cancer patients with newly diagnosed or progressive brain metastatic lesions.

For HER2-negative breast cancer patients, there are also multiple pertinent studies. NCT05953168 explores the efficacy and safety of T-DXd as a first-line treatment for locally advanced or metastatic TNBC and HER2 low-expressing intraductal androgen receptor subtypes. DESTINY-Breast06 (NCT04494425) evaluates the comparative efficacy of T-DXd *versus* physician’s choice of chemotherapy for HER2 low-expressing, hormone receptor-positive breast cancer patients who progress after metastatic endocrine therapy. Furthermore, DESTINY-Breast15 (NCT05950945) is a phase III clinical trial aiming to comprehensively investigate the role of T-DXd in unresectable and/or metastatic HR-negative/positive breast cancer with HER2 low or HER2 immunohistochemistry (IHC) of 0.

#### 6.1.3 SG

In the case of HER2-negative breast cancer, the NCT04595565 trial allocated patients with residual lesions post-neoadjuvant chemotherapy into two groups: one receiving SG and the other receiving the treatment of the physician’s choice (TPC) in a 1:1 ratio. The interim safety analysis from the SASCIA study (NCT04595565) was reported at ESMO Breast 2022 in May 2022. As of May 2022, out of 230 patients screened for enrollment, 88 were used for interim safety analysis (45 in the SG arm and 43 in the TPC arm). The incidence of grade 3–4 adverse events was 66.7% in the SG group and 20.9% in the TPC group. More hematological and non-hematological toxicities were reported in the SG arm, and more dose delays were observed compared to the TPC (Cape) arm. Nevertheless, the overall safety was deemed manageable (Marmé et al., 2022). Another study, NCT04647916, explores the role of SG in treating patients with HER2-negative breast cancer (with brain metastases).

The TROPiCS-02 trial (NCT03901339), targeting HR+/HER2-metastatic breast cancer, screened 776 patients from May 2019 to April 2021. Patients were randomly assigned to receive SG therapy (*n* = 272) or chemotherapy (*n* = 271). Upon achieving the primary endpoint, there was a 34% reduction in the risk of progression or death (hazard ratio, 0.66 [95% CI, 0.53 to 0.83; *p* = 0.0003]). The median progression-free survival (PFS) was 5.5 months for the SG group and 4.0 months for the chemotherapy group. Notable grade 3 adverse events associated with the treatment were neutropenia (51% SG vs. 38% chemotherapy) and diarrhea (9% SG vs. 1% chemotherapy) (Rugo et al., 2022a). An ongoing Asian study of SG (NCT04639986) is focused on HR+/HER2-metastatic breast cancer patients.

As for TNBC, the NCT05382299 trial involves patients who are PD-L1 negative or those previously treated with anti-PD-(L)1 agents that express PD-L1. The primary objective is to compare the PFS of SG *versus* TPC in participants with previously untreated, locally advanced, inoperable, or metastatic TNBC. While PD-L1 inhibitors are effective in TNBC, they are most efficient and tend to develop resistance in PD-L1-positive patients, whereas PD-L1 expression is generally low in TNBC. Thus, understanding the role of SG in the early treatment of PD-L1-negative TNBC can contribute to new treatment approaches. The Ever-132-001 study (NCT04454437), a single-arm, phase IIb trial involving Chinese mTNBC patients who had failed ≥2 previous chemotherapy regimens, is also underway. As of 6 August 2021, 80 Chinese female patients (median age 47.6 years; range 24–69.9 years) had received ≥1 dose of SG with a median of 8 cycles of treatment. The median PFS was 5.55 months. The most common grade ≥3 treatment-emergent adverse events (TEAE) were decreased neutrophil count (62.5%), decreased white blood cell count (48.8%), and anemia (21.3%). A total of 6.3% discontinued SG due to TEAE (96).

### 6.2 Combination medication

#### 6.2.1 T-DM1

Combination with Chemotherapy: Breast cancer chemotherapy encompasses a diverse range of drugs, primarily including anthracyclines, taxanes, alkylating agents, platinum compounds, and gemcitabine. In the NCT02562378 trial, researchers evaluated the combination of T-DM1 and non-pegylated liposomal doxorubicin (NPLD)—a liposomal anthracycline drug that exhibits similar antitumor efficacy to traditional anthracyclines but significantly reduces cardiotoxicity—for its dose-limiting toxicity and maximum tolerated dose (MTD) in HER2-positive metastatic breast cancer. This study primarily targeted patients initially treated with anthracycline drugs who showed disease progression after treatment with trastuzumab and taxane drugs. The findings indicated that while the combination of NPLD and T-DM1 is feasible, this regimen does not seem to enhance the therapeutic effect of T-DM1 in patients with HER2-positive metastatic breast cancer (López-Miranda et al., 2020). In another clinical trial, NCT03190967, for HER2-positive breast cancer patients who developed brain metastases after local treatment, researchers explored the potential of oral TMZ combined with T-DM1 for systemic disease control as a secondary preventive strategy for these patients (Zimmer et al., 2020).

Combination with TKIs: Tyrosine Kinase Inhibitors (TKIs) are a class of small molecule inhibitors that target tyrosine kinases. They function by competitively binding to the ATP-binding site of the kinase, inhibiting its activity, and subsequently interrupting downstream signaling pathways. Within the therapeutic strategies for breast cancer, TKIs are primarily employed for HER2-positive breast cancers. T-DM1 delivers the drug into cells by binding to the external domain of the HER2 receptor, while TKIs block the activity of the internal kinase domain of HER2. This ensures effective inhibition of the HER2 receptor, both externally and internally. Additionally, TKIs have potential advantages against the truncated HER2 variant (p95HER2), which lacks the binding sites for trastuzumab and pertuzumab. This variant is present in up to 30% of HER2-positive metastatic breast cancers. Thus, the combination of TKIs with T-DM1 offers a more potent, comprehensive, and targeted treatment option for breast cancer patients, especially those who have developed resistance to traditional HER2-targeted therapies (Scaltriti et al., 2010). NCT02236000 is a 3 + 3 dose-escalation study targeting metastatic breast cancer patients who progressed after treatment with trastuzumab, pertuzumab, and taxanes. The study explored the safety, tolerability, and efficacy of combined treatment with T-DM1 and neratinib. A total of 27 patients were treated with T-DM1 3.6 mg/kg intravenously every 3 weeks and escalating doses of oral neratinib. The trial results showed some dose-limiting toxicities, including grade 3 diarrhea and nausea, as well as other grade 3–4 toxicities. Of the 19 evaluable patients, 12 (63%) exhibited objective responses (Abraham et al., 2019). NCT02236000 is a 3 + 3 dose-escalation study targeting metastatic breast cancer patients who progressed after treatment with trastuzumab, pertuzumab, and taxanes. The study explored the safety, tolerability, and efficacy of combined treatment with T-DM1 and neratinib. A total of 27 patients were treated with T-DM1 3.6 mg/kg intravenously every 3 weeks and escalating doses of oral neratinib. The trial results showed some dose-limiting toxicities, including grade 3 diarrhea and nausea, as well as other grade 3–4 toxicities. Of the 19 evaluable patients, 12 (63%) exhibited objective responses [110]. The NCT01983501 clinical trial demonstrated that the combination of tucatinib and T-DM1 exhibited acceptable toxicity and preliminary antitumor activity in heavily pretreated ERBB2/HER2-positive metastatic breast cancer patients (Borges et al., 2018). Two phase III clinical trials, NCT03975647 and NCT04457596, aim to compare the combination of tucatinib and T-DM1 with T-DM1 monotherapy in patients with inoperable locally advanced or metastatic HER2-positive breast cancer and in patients with residual disease post-neoadjuvant and HER2-targeted treatment (O Sullivan et al., 2021).

Combination with CDK4/6 Inhibitors: Within the cell cycle, CDK4/6 binds to cyclin D, facilitating the transition of cells from the G1 to the S phase, enabling DNA replication. By inhibiting the activity of CDK4/6, these inhibitors can prevent the proliferation and growth of tumor cells. CDK4/6 inhibitors are primarily used for hormone receptor positive, HER2-negative advanced breast cancer. The FDA has approved certain CDK4/6 inhibitors in combination with aromatase inhibitors as a first-line endocrine therapy for postmenopausal women, or in combination with fulvestrant as a first or second-line treatment. The combination of T-DM1 and CDK4/6 inhibitors can delay disease progression due to resistance in HER2-positive breast cancer (Goel et al., 2016). Moreover, since cyclin D1 is downstream of HER2 signaling (Yu et al., 2001), CDK4/6 inhibitors can enhance the therapeutic effect on HER2-positive breast cancer. NCT02657343, titled “An Open-Label, Phase Ib/II Clinical Trial Of CDK 4/6 Inhibitor, Ribociclib (Lee011), In Combination With Trastuzumab Or T-Dm1 For Advanced/Metastatic HER2-Positive Breast Cancer,” overall, the safety profile of this combination therapy is favorable, but its efficacy requires further investigation (Goel et al., 2019).

Combining Immune Checkpoint Inhibitors: PD-1 is predominantly expressed on activated T cells, B cells, NK cells, activated monocytes, and dendritic cells. Its ligand, PD-L1, is primarily located on the surface of tumor cells. Under physiological conditions, PD-L1 binds to PD-1 on the surface of T cells, regulating immune homeostasis by inhibiting T cell activation and inducing T cell apoptosis, thereby preventing over-activation of the immune system. Tumor cells expressing PD-L1 interact with PD-1, inhibiting T cell migration, proliferation, and the secretion of anti-tumor cytokines, consequently weakening the body’s anti-tumor immunity (Zhu et al., 2021). Preclinical studies have indicated that the combination of T-DM1 with immune checkpoint inhibitors targeting PD-1 and cytotoxic T-lymphocyte-associated antigen 4 can induce tumor regression (Müller et al., 2015). Results from the NCT02924883 (KATE2) trial suggest that T-DM1 combined with atezolizumab may hold potential for further research in the PD-L1 positive subgroup, but the benefits in the overall patient population are not evident (Emens et al., 2020). KATE3 (NCT04740918) is a phase III clinical trial aiming to further evaluate the efficacy and safety of this combination in HER2-positive, PD-L1 positive locally advanced or metastatic breast cancer patients who have previously received trastuzumab (optionally pertuzumab) and taxane treatments. NCT04873362 is a phase III randomized, double-blind, placebo-controlled clinical trial designed to assess the efficacy and safety of atezolizumab or placebo combined with T-DM1 in HER2-positive breast cancer patients at high risk of recurrence after neoadjuvant treatment (Hurvitz et al., 2022). NCT03032107 is a phase Ib clinical trial focusing on the effects of the combination treatment of T-DM1 and pembrolizumab in metastatic HER2-positive breast cancer patients who have previously been treated with taxane, trastuzumab, and pertuzumab but have not experienced T-DM1. The study results indicate that this combination treatment is safe and well-tolerated (Waks et al., 2022).

#### 6.2.2 T-DXd

Combining Monoclonal Antibody Drugs: Both trastuzumab and pertuzumab are monoclonal antibodies targeting HER2. Trastuzumab, by binding to the HER2 receptor, effectively disrupts its signaling pathway, subsequently inhibiting the proliferation, division, and spread of cancer cells. On the other hand, pertuzumab binds to a different domain of HER2, preventing its interaction with other proteins in the HER family, thereby further suppressing aberrant signaling. The combined treatment with these two monoclonal antibodies offers a synergistic approach to treating HER2-positive breast cancer, providing patients with a more comprehensive treatment option. Currently, the ESTINY-Breast09 trial (NCT04784715) is evaluating the efficacy and safety of T-DXd in combination with pertuzumab as a first-line treatment regimen for HER2-positive metastatic breast cancer.

Combining TKIs: The study NCT04539938 is investigating the combined treatment of tucatinib and T-DXd for locally advanced or metastatic HER2-positive breast cancer in patients who have previously received treatment.

Additionally, the DESTINY-Breast07 (NCT04538742) and DESTINY-Breast08 (NCT04556773) phase I/II clinical trials are respectively exploring the safety and efficacy of T-DXd combined with different drugs for the treatment of metastatic breast cancer that is either HER2-positive or has low HER2 expression.

#### 6.2.3 SG

Combination with PARP inhibitors: The most notable gene mutation associated with breast cancer is the germline BRCA mutation. Germline BRCA mutations, inherited from germ cells and passed onto the next-generation, have been associated with a clear familial propensity for ovarian cancer (Li et al., 2022). BRCA1 and BRCA2, two critical oncogenes, are located on chromosomes 17 and 13 of the human cell nucleus, respectively. These genes and PARPase each regulate a DNA repair pathway. If cancer cells harbor BRCA1/2 gene mutations and another DNA repair pathway is impeded by PARPase inhibitors, such as Olaparib, then both DNA repair pathways are blocked, leading to cancer cell death due to untimely DNA repair. In contrast, normal cells, having intact BRCA proteins, can still repair DNA relatively normally even after exposure to PARP inhibitors and thus, survive (Tutt and Ashworth, 2002; Tutt et al., 2021). SG, exerting its anticancer effects by interfering with DNA synthesis, theoretically, could increase the efficacy when combined with PARP inhibitors. The NCT04039230 trial will investigate the effects of SG in combination with Talazoparib in patients with metastatic triple-negative breast cancer.

Combination with immune checkpoint inhibitors: PD-1/PD-L1 immunosuppressants have been employed in various cancer treatments, and Pembrolizumab has shown significant efficacy against TNBC in the KEYNOTE-522 trial (Schmid et al., 2022). Several trials are studying the potential of SG in combination with Pembrolizumab: the NCT04468061 trial focuses on PD-L1-negative metastatic triple-negative breast cancer, the NCT05382286 trial aims to compare SG plus Pembrolizumab with treatment of choice (TPC) plus Pembrolizumab in terms of progression-free survival (PFS) in patients with previously untreated PD-L1-positive locally advanced inoperable or metastatic triple-negative breast cancer, and the NCT04434040 trial investigates the potential of SG and atezolizumab combination in preventing TNBC recurrence. In patients with metastatic HR+/HER2-breast cancer, the NCT04448886 trial is assessing the safety and efficacy of SG, with or without Pembrolizumab. Given that immune checkpoint inhibitors tend to develop resistance, the addition of SG could help evade this challenge. The NCT05675579 trial is currently evaluating the safety and efficacy of pre-surgical SG and Pembrolizumab administration in early-stage triple-negative breast cancer resistant to immunotherapy (Relevant clinical trials are organized in Table 3).

**TABLE 3:** Clinical trials of Combination medication.

Types of combination	NCT Number/Phase	Conditions	Interventions	Primary completion
T-DM1				
chemotherapy	NCT03190967/Ⅰ, Ⅱ (Zimmer et al., 2020)	Secondary Prevention of HER2+ BC Brain Metastases Following Stereotactic Radiosurgery	E:T-DM1	28 June 2021
			C:T-DM1 plus TMZ	
	NCT02562378/Ⅰ	HER2+ MBC previously treated with taxanes and trastuzumab-based therapy	E = T-DM1+non-pegylated liposomal doxorubicin	December 2018
TKI	NCT02236000/Ⅰ/Ⅱ (Abraham et al., 2019)	Women With HER2+MBC	E = T-DM1 and Neratinib	2020.10
			C = T-DM1 or Neratinib	
	NCT01983501/Ⅰ (Borges et al., 2018)	HER2+ BC	E = T-DM1+ Tucatinib	10 October 2017
	NCT03975647/Ⅲ (HER2CLIMB-02) (O Sullivan et al., 2021)	HER2+Unresectable Locally-A/MBC	E = Tucatinib + T-DM1	29 June 2023
			C=Placebo + T-DM1	
	NCT04457596/Ⅲ	high risk patients with HER2+ BC and residual disease after neoadjuvant HER2-directed therapy	E = T-DM1 + tucatinib	January 2028
			C = T-DM1 + placebo	
CDK4/6 inhibitors	NCT02657343/Ⅰ/Ⅱ (Goel et al., 2019)	HER2+, ABC/MBC	E = Ribociclib + T-DM1	27 August 2020
			E = Ribociclib + Trastuzumab	
			E = Ribociclib + Trastuzumab + Fulvestrant	
immune checkpoint inhibitor	NCT03032107/Ⅰ (Waks et al., 2022)	HER2+ MBC	E = T-DM1+ Pembrolizumab	29 October 2020
	NCT04740918/Ⅲ (KATE3)	HER2+ and PD-L1-Positive Locally A/MBC Who Have Received Prior Trastuzumab- (+/- Pertuzumab) and Taxane-Based Therapy	E = T-DM1 + Atezolizumab	31 December 2023
			C = T-DM1 + Placebo	
	NCT04873362/Ⅲ (Hurvitz et al., 2022)	HER2+ BC at High Risk of Recurrence Following Preoperative Therapy	E = Atezolizumab + T-DM1	25 June 2026
			C=Placebo + T-DM1	
PI3K inhibitor	NCT02038010/Ⅰ	HER2+ MBC With Progression on Prior Trastuzumab and Taxane-Based Therapy	E = T-DM1+ BYL719	15 April 2015
T-DXd				
monoclonal antibody	NCT04784715/Ⅲ (DESTINY-Breast09)	HER2+ MBC	EA = T-DXd + Placebo	28 March 2025
			EB = T-DXd + Pertuzumab	
			C=Taxane + Pertuzumab + Trastuzumab	
TKI	NCT04539938/Ⅰ/Ⅱ	Previously Treated HER2+ Unresectable Locally-A/MBC	E: Tucatinib + T-DXd	31 January 2024
Multiple types of combination medications	NCT04538742/Ⅰ/Ⅱ (DESTINY-Breast07)	HER2+ MBC	E A: T-DXd + Durvalumab	31 January 2025
			EB: T-DXd + Pertuzumab	
			EC: T-DXd + Paclitaxel	
			ED: T-DXd + Durvalumab + Paclitaxel	
			EE: T-DXd	
			E F: T-DXd + Tucatanib	
			E G: T-DXd + Tucatinib (Part 2 Only)	
			E H: T-DXd (Part 2 Only)	
	NCT04556773/Ⅰ/Ⅱ (DESTINY-Breast08)	HER2-low MBC	E A: T-DXd + capecitabine	16 August 2023
			E B: T-DXd + durvalumab + paclitaxel	
			E C: T-DXd + capivasertib	
			ED: T-DXd + anastrozole	
			E E: T-DXd + fulvestrant	
SG				
PAPR inhibitors	NCT04039230/Ⅰ/Ⅱ	MBC	E = SG + Talazoparib	December 2023
immune checkpoint inhibitor	NCT05675579/Ⅱ	Immunochemotherapy-resistant Early-stage TNBC	E = SG + Pembrolizumab	March 2024
	NCT05633654/Ⅲ	TNBC with residual invasive disease after surgery and neoadjuvant therapy	E = SG + Pembrolizumab	July 2027
			C=Capecitabine or Pembrolizumab + Capecitabine	
	NCT04468061/Ⅱ	MTNBC and PD-L1-	E = SG + Pembrolizumab	January 2024
			E = SG	
	NCT05382286/Ⅲ	Previously Untreated, Locally Advanced Inoperable or MTNBC and PD-L1 +	E = SG + Pembrolizumab	February 2027
			C= Pembrolizumab + Paclitaxel/nab-Paclitaxel/Gemcitabine + Carboplatin	
	NCT04230109/Ⅱ (NeoSTAR)	Invasive Breast Cancer; TNBC; ER-;PR-;HER2-	E = SG E = SG + Pembrolizumab	October 2025
	NCT04434040/Ⅱ (ASPRIA)	Breast Cancer; TNBC; Residual Cancer Circulating Tumor DNA	E = SG + Atezolizumab	December 2023
	NCT03971409/Ⅱ	Stage III -Stage IV Breast Cancer; Invasive Breast Carcinoma	E = Avelumab + Binimetinib/Anti-OX40 Antibody PF-04518600/Utomilumab/Liposomal Doxorubicin/SG	July 2023
		Recurrent Breast Carcinoma		
		TNBC		
		Unresectable Breast Carcinoma		
CDK4/6 inhibitors	NCT05113966/Ⅱ	MTNBC Who Received at Least Two Prior Treatments, at Least One in the Metastatic Setting	E = Trilaciclib + SG	June 2023
PI3K inhibitors	NCT05143229/Ⅰ	BC	E = SG + Alpelisib	July 2023
CD47 monoclonal antibody	NCT04958785/Ⅱ	Unresectable, Locally -A/MTNBC	E = Magrolimab + Nab-Paclitaxel/Paclitaxel	August 2024
			C= Nab-Paclitaxel/Paclitaxel	
			E = Magrolimab + SG	
Multiple randomized drug therapy combinations	NCT03424005/Ⅰ/Ⅱ	MBC	Drug: Capecitabine/Atezolizumab/Ipatasertib/SGN-LIV1A/Bevacizuma/Chemotherapy (Gemcitabine + Carboplatin or Eribulin)/Selicrelumab/Tocilizumab/Nab-Paclitaxel/SG	May 2024

### 6.3 Additional clinical trials

#### 6.3.1 T-DM1

For HER2-positive breast cancer, commonly used neoadjuvant chemotherapy regimens include anthracycline and taxane-based treatments, supplemented with HER2-targeted therapies such as trastuzumab and/or pertuzumab. Neoadjuvant dual human epidermal growth factor receptor 2 (HER2) blockade therapy, utilizing trastuzumab and pertuzumab in combination with paclitaxel, has achieved an overall pathological complete response (pCR) rate of 46%. Meanwhile, T-DM1 combined with lapatinib and albumin-bound paclitaxel has demonstrated efficacy in patients with metastatic HER2-positive breast cancer. Therefore, the NCT02073487 trial compared the neoadjuvant treatment effects of these two regimens in early-stage HER2-positive breast cancer patients. Among 30 evaluable patients, the experimental group (T-DM1 combination) showed 100% of patients with a residual cancer burden (RCB) of 0 or I, compared to 62.5% in the standard treatment group, a significant difference (*p* = 0.0035). Notably, in the ER-positive subgroup, all patients in the experimental group achieved RCB 0-I, while only 25% in the standard group did (*p* = 0.0035). Adverse events were similar between the two groups. Thus, for early-stage HER2-positive breast cancer, neoadjuvant treatment with T-DM1, lapatinib, and nab-paclitaxel is more effective than standard treatment, especially in the ER-positive patient population (Patel et al., 2019). The FDA has not yet approved T-DM1 for preoperative use in breast cancer but has approved it for other breast cancer indications. The FDA has approved pertuzumab for preoperative treatment. The NCT02326974 trial, targeting early-stage HER2-positive breast cancer patients, investigated the effects of T-DM1 combined with Pertuzumab in neoadjuvant treatment. Results showed a pCR rate of 54.6% in patients with non-heterogeneous HER2 amplification (data from ClinicalTrials.gov) and 0% in those with heterogeneous HER2 amplification. Additionally, treatment-related adverse events mainly included fatigue, anemia, and diarrhea. The NCT02568839 trial, conducted at nine locations in Sweden with 202 participants, compared the efficacy of two neoadjuvant treatment methods for ERBB2-positive breast cancer. The primary objective was to compare the pCR rate of the standard neoadjuvant treatment combination of docetaxel, trastuzumab, and pertuzumab (DTP) with T-DM1 monotherapy in ERBB2-positive breast cancer patients. Results indicated a pCR rate of 45.5% for the standard group and 43.9% for the experimental group, with no statistically significant difference between them. This suggests that for selected ERBB2-positive breast cancer patients, neoadjuvant treatment can be de-escalated to T-DM1 monotherapy without compromising efficacy (Hatschek et al., 2021).

T-DM1 Cardiotoxicity Study: The SAFE-HEaRt study (NCT01904903) is the first prospective trial aimed at assessing whether HER2-targeted therapies can be safely administered to patients with a slight reduction in cardiac function, under the backdrop of ongoing cardiac treatment and monitoring. Of the 31 enrolled patients, 30 were evaluable. The study results indicated that the average LVEF was 45% before treatment and 46% after. Notably, 90% of the patients (27 individuals) successfully completed the planned HER2-targeted treatment. Only two patients experienced cardiac events, with one patient having an asymptomatic LVEF deterioration to ≤35%. These findings suggest that, with cardioprotective medications and close cardiac monitoring, HER2-targeted treatment is safe in breast cancer patients, even if their LVEF is slightly reduced (Lynce et al., 2019).

#### 6.3.2 T-DXd

The objective of the ARIADNE clinical trial (NCT05900206) is to compare the effects of T-DXd with standard preoperative treatment in patients with non-metastatic HER2-positive breast cancer. The trial aims to address two primary questions: firstly, whether T-DXd is more effective than the standard preoperative treatment; and secondly, whether biomarkers can be identified in the patient’s tumor or blood to predict treatment responses. Participants will be divided into two groups, with one group receiving three cycles of T-DXd treatment and the other receiving three cycles of standard care. Subsequent treatments will be determined based on the molecular subtype of the tumor. The purpose of this study is to offer new possibilities for improving the treatment of patients with HER2-positive breast cancer.

#### 6.3.3 SG

The NCT05552001 trial aims to evaluate the safety and efficacy of SG in patients with metastatic triple-negative breast cancer. The trial employs biomarker analysis to identify markers that can predict either response to or resistance against the drug.

On the other hand, the NCT05332561 trial adopts a precision oncology strategy for enrolling participants, primarily those with early-stage breast cancer types such as TNBC, HER2+, and HR+/HER2-. Patients are assigned to treatment groups based on an in-depth molecular characterization of their tumors as part of the COGNITION registry program, which constitutes a seven-arm umbrella clinical trial. Arms 1-6 are administered Atezolizumab, Inavolisib, Ipatasertib, Olaparib, SG, and Trastuzumab/pertuzumab respectively, while arm 7 does not receive any intervention. For arm 5 (SG), the required biomarker is TROP-2 overexpression (verified with IHC and excluding known/reported homozygous polymorphism in UGT1A1*28) (Relevant clinical trials are organized in Table 4).

**TABLE 4:** Other clinical trials.

NCT Number/Phase	Study title	Conditions	Interventions	Primary completion
T-DM1				
NCT02073487/Ⅱ (Patel et al., 2019)	Randomized Open Label PhII Trial of Neoadjuvant Trastuzumab Emtansine (Te) in Combination w/Lapatinib(L) Followed by Abraxane (A) Compared w/Trastuzumab Plus Pertuzumab Followed by Paclitaxel in Her2/Neu Over-Expressed Breast Cancer Patients	Her2/Neu Over-Expressed Breast Cancer	E = T-DM1 + Lapatinib + Abraxane C = Trastuzumab + Pertuzumab + Paclitaxel	January 2019
NCT02326974/Ⅱ (Hatschek et al., 2021)	The Impact of HER2 Heterogeneity on the Treatment of Early-stage HER2-positive Breast Cancer: a Phase II Study of T-DM1 in Combination With Pertuzumab in the Preoperative Setting	Early-stage HER2-positive BC	E = T-DM1 and Pertuzumab Excision of tumor/mastectomy of biopsy residual tumor within 42 days of the last cycle of therapy	June 2018
NCT01904903/Ⅱ (Lynce et al., 2019)	SAFE-HEaRt: A Pilot Study Assessing the Cardiac SAFEty of HER2 Targeted Therapy in Patients With HER2 Positive Breast Cancer and Reduced Left Ventricular Function	HER2-positive BC and Reduced Left Ventricular	HER2 therapies: Trastuzumab/Pertuzumab/T-DM1 cardiac medications: beta-blockers and ACE-inhibitors	June 2019
NCT02568839/Ⅱ/Ⅲ	PREDIX HER2 - Neoadjuvant Response-guided Treatment of HER2 Positive Breast Cancer. Part of a Platform of Translational Phase II Trials Based on Molecular Subtypes	Early-Stage Breast Carcinoma HER-2 Positive Breast Cancer	E = docetaxel + trastuzumab + pertuzumab	February 2019
			C = T-DM1	
NCT05429684/Ⅲ	Precise Targeted Therapy for Refractory HER2 Positive Advanced Breast Cancer Based on Genome Signature and Drug Sensitivity of PDO Model	A:HER2 low expression	A:Trastuzumab/Pertuzumab/Nab paclitaxel	28 February 2024
		B.:HER2 amplified	B:Trastuzumab/Pertuzumab/Nab paclitaxel	
		C.:HER2 mutation	C.:Pyrotinib/Capecitabine	
		D:HER2 downstream mutation	D:Trastuzumab/Nab paclitaxel/T-DM1/Everolimus	
		E:Hormone receptor pathway activation	E:Trastuzumab/CDK4/6 inhibitor/AI	
		F:Immune activation	F:Trastuzumab/Anti-PD-1 monoclonal antibody	
T-DXd				
NCT05900206/Ⅰ/ⅡARIADNE	A Randomized Trial of Trastuzumab Deruxtecan and Biology-Driven Selection of Neoadjuvant Treatment for HER2-positive Breast Cancer	HER2-positive Breast Cancer	E = T-DXd	1 June 2026
			C=Standard treatment (TCHP or PCHP)	
SG				
NCT05552001/Ⅲ	ISIdE: Open Label, Multicentric, Single-arm Phase IIIB Trial to Evaluate the Safety and Efficacy of Sacituzumab Govitecan in Triple Negative Metastatic Breast Cancer Patients With a Biomarker Analysis	TNBC; Metastatic Breast Cancer	E = SG	July 2024
NCT05520723/Ⅱ	Preventive stRategy for IMMU132-relatED AEs in TNBC - PRIMED	TNBC; Breast Cancer	E = SG+ + Loperamide + G-CSF	December 2025
NCT05332561/Ⅱ	Genomics Guided Targeted Post-neoadjuvant Therapy in Patients With Early Breast Cancer (COGNITION-GUIDE)	Early-stage Breast Cancer	E = Atezolizumab/Inavolisib/Ipatasertib/Olaparib/SG/Trastuzumab/pertuzumab	April 2029

## 7 Major adverse drug reactions

The mechanism underlying the adverse reactions of ADCs (Antibody-Drug Conjugates) is complex, involving its constituent parts: mAb, payload, linker, and conjugation process. The choice of mAb determines target specificity and potential off-target binding, which may lead to off-target adverse reactions. The cytotoxicity of the payload might result in adverse reactions associated with specific payload types, such as neuropathy and thrombocytopenia. The stability of the linker determines where the payload is released, potentially causing target-specific or widespread adverse reactions. The conjugation process of the ADC determines the actual drug-to-antibody ratio (DAR), and heterogeneity in this ratio might affect therapeutic efficacy and increase adverse reactions. In summary, each component of the ADC can influence its safety and efficacy (Donaghy, 2016; Breast Cancer Group BoO, 2022).

The primary adverse events of T-DM1 are thrombocytopenia and peripheral neuropathy, a typical toxicity of microtubule inhibitor-conjugated ADCs (Krop et al., 2010; Burris et al., 2011; Verma et al., 2012). A recent safety analysis of 884 patients exposed to T-DM1 revealed common adverse reactions including fatigue (46.4%), nausea (43%), thrombocytopenia (32.2%), headache (29.4%), and constipation (26.5%). Notably, grade 3 or higher thrombocytopenia occurred in 44.4% of Asian patients, significantly higher than in non-Asian patients (10.6%). Grade ≥3 thrombocytopenia rarely correlated with grade ≥3 bleeding events, but some fatal severe bleeding cases related to thrombocytopenia were documented in over 4200 patients exposed to T-DM1 (Dieras et al., 2014). To ensure patient safety, it is recommended to closely monitor patients with thrombocytopenia, check platelet counts before each administration, and closely watch those with reduced platelets or those on anticoagulant therapy. In most cases, thrombocytopenia can recover after treatment pause, dose reduction, or discontinuation. Symptomatic supportive treatments, including platelet transfusion and thrombopoietin-receptor agonists, can be applied when needed (Breast Cancer Group BoO, 2022). A retrospective study including 6 cases of breast cancer patients with dose reductions and treatment delays due to thrombocytopenia secondary to T-DM1 or T-DXd therapy was reported to show efficacy in the treatment of immune thrombocytopenia after the use of thrombopoietin receptor agonists (TPO-RA) such as eltrombopag, romiplostim, and avatrombopag (Rainone et al., 2023a).

In the DESTINY-Breast 01, 02, 03, and 04 clinical trials, the primary adverse events of T-DXd were nausea and vomiting, as well as ILD/pneumonia. The incidence rates of nausea were 69.0%, 65.0%, 70.0%, and 68.0%, respectively, while those for vomiting were 24.0%, 25.0%, 23.0%, and 24.0%. For the prevention of nausea and vomiting, pharmacological prophylaxis, such as antiemetic drugs recommended by the NCCN Clinical Practice Guidelines, and lifestyle adjustments, such as avoiding foods and odors that might trigger nausea and vomiting, can be adopted. The incidence rates of ILD/pneumonia were 13.6%, 14.0%, 15.0%, and 13.0% (Rugo et al., 2022b). T-DXd is an anti-HER-2 ADC drug associated with a higher incidence of ILD, with ILD-related mortality rates ranging between 1.7% and 2.2%. Most T-DXd-induced ILD cases appear within the first year after treatment, especially evident in Asian breast cancer or solid tumor patients. To manage this side effect, it’s recommended to regularly conduct complete blood counts, radiological examinations, and high-resolution CT (HRCT) scans on patients to detect and address early signs of ILD. Invasive tests like bronchoalveolar lavage might be required when history and auxiliary examinations cannot confirm the diagnosis. Additionally, pulmonary function tests and blood oxygen measurements are essential tools for assessment and monitoring (Breast Cancer Group BoO, 2022). ILD is a complex process involving suppression of inflammation and prevention of fibrosis, and its treatment requires multidisciplinary management. In grade 1 ILD, T-DXd is first discontinued and subsequently continued or discontinued depending on health status; in grade 2 ILD, T-DXd is permanently discontinued, and systemic prednisolone therapy is instituted and closely monitored; in grades 3 and 4 ILD, T-DXd is permanently discontinued, and high-dose methylprednisolone is administered intravenously for 3 days immediately, followed by prednisone at a dose of ≥1 mg/kg/day, until the clinical and chest CT findings completely subside, during which time other immunosuppressive agents may be considered depending on the patient’s condition (Swain et al., 2022; Wekking et al., 2023).

SG is an antibody-drug conjugate consisting of a humanized anti-Trop-2 monoclonal antibody (hRS7) and SN-38, the active metabolite of irinotecan (Goldenberg et al., 2015). The toxicity associated with SG is primarily related to SN-38, but its overall incidence and severity are much lower than those observed for irinotecan (Bardia et al., 2017). Data from the ASCENT trial reveal a higher incidence of adverse reactions in the SG group compared to the chemotherapy group. The most frequent adverse reactions of all grades in the SG group included neutropenia (63%), diarrhea (59%), nausea (57%), alopecia (46%), fatigue (45%), and anemia (34%). The most common Grade ≥3 adverse reactions were primarily neutropenia (51%), leukopenia (10%), and diarrhea (10%). These findings are in line with the interim safety analysis of the SASCIA (NCT04595565) trial. Notably, there were no instances of death or serious cardiovascular toxicity attributed to SG.

For the management of common adverse reactions associated with SN-38, pharmacologic countermeasures are summarized in a review by Bailly, C., as shown in Table 5, which provides a reference for the prevention of SG-related adverse reactions. Early-onset diarrhea may be associated with acute cholinergic syndrome and may be managed with some anticholinergic agents. The pathogenesis of late-onset diarrhea is more complex and can be addressed with oral alkalizing agents, herbal supplements, and probiotics (Bailly, 2019).The NCT05520723 trial will evaluate the effectiveness of loperamide and G-CSF for managing diarrhea and neutropenia, respectively, associated with SG. The effects of UGT1A1 polymorphisms impedes the detoxification of SN-38, and patients with UGT1A1 *28/*28 genotypes have a higher likelihood of experiencing grade ≥3 adverse reactions (ADRs) compared to patients with the genotypes of 1/*28 and *1/*1 (Bardia et al., 2021b; Spring et al., 2021; Rugo et al., 2022c). Therefore, UGT1A1 genetic testing of patients to personalize therapy such as adjusting the dose administered has the potential to reduce the incidence of ADRs. In addition, drugs that inhibit UGT1A1 enzyme activity, such as pazopanib and erlotinib, should be avoided in combination with SG.

**TABLE 5:** Drugs with potential efficacy against the major adverse effects of ADCs.

ADR		Drugs	
thrombocytopenia (Rainone et al., 2023a)		thrombopoietin receptor agonists (TPO-RA):eltrombopag, romiplostim, and avatrombopag	
ILD (Wekking et al., 2023; Swain et al., 2022)		Immunosuppressants: infliximab, mycophenolate mofetil	
Diarrhoea (Bailly, 2019)	Early onset diarrhoea	Prophylactic use of atropine sulphate, hyoscyamine, scopolamine bu-tylbromide	
	Late-onset diarrhoea	Oral alkalizing drugs	magnesium oxide and sodium hydrogen carbonate
		small molecule drugs	TCH-3562, amoxapine, ciprofloxacin, darunavir
		plant extracts	the Gegen Qinlian decoction, the 4-herb formulation PHY906, the HuangQin decoction, the Shengjiang Xiexin decoction, the Banxia Xiexin decoction, Kampo medicine Hangeshashin-to (TJ-14)
		probiotics	UFMG A-905 (Sc-905), Colon Dophilus™
Neutropenia (Bailly, 2019)		Alkaline combination of ursodeoxycholic acid、magnesium oxide and sodium hydrogen carbonate	

Quality of life assessments from the ASCENT trial, using health-related quality of life (HRQoL) questionnaires, indicated that patients in the SG group had better HRQoL scores than those in the TPC group (Loibl et al., 2022). Overall, most of the ADRs observed in mTNBC patients treated with SG can be managed through symptomatic treatment, dose reduction, and delayed dosing. This points to a manageable safety profile that can enhance the quality of life for patients.

## 8 Discussion and outlook

Breast cancer treatment is markedly heterogeneous, firstly due to the fact that it is a heterogeneous disease involving genetic and environmental factors, and secondly it is resistant to cytotoxic drugs and conventional targeted therapies (Barzaman et al., 2020; Mehraj et al., 2021). Three marketed antibody-drug conjugates (ADCs) can be used for the treatment of essentially most types of breast cancer, especially some drug-resistant, refractory metastatic advanced breast cancers. ADC combination therapies may enhance each other’s activity, helping to reduce the risk of drug resistance and further improve efficacy, and several clinical trials are underway, but it is worth noting that toxicities and drug-drug interactions of ADC drugs should not be ignored (Drago et al., 2021; Dumontet et al., 2023).

Globally, T-DM1 has demonstrated a favorable safety and efficacy profile in both early- and late-stage HER2-positive patient populations, but in Asia, T-DM1 has been associated with a higher rate of thrombocytopenia than in non-Asian populations (Wuerstlein et al., 2022; Rainone et al., 2023b). It is especially outstanding in patients who are resistant after traditional adjuvant or neoadjuvant treatment, still have residuals, relapse, or have brain metastases. However, it is primarily used as a second-line treatment. Compared to the traditional docetaxel plus carboplatin regimen, the traditional regimen shows a higher pCR rate in the neoadjuvant treatment phase, but the T-DM1 regimen demonstrates comparable efficacy and better safety in the adjuvant treatment phase (de Haas et al., 2023). Therefore, the exploration of T-DM1 as a first-line treatment for breast cancer remains to be done. However, long-term use of T-DM1 can also lead to resistance. Its resistance mechanism involves intracellular metabolic disorders (Ríos-Luci et al., 2017), activation of STAT3 in signal transduction (Wang et al., 2018), specific tumor microenvironment factors (Endo and Wu, 2021), and overexpression of Polo-like kinase 1 (PLK1) (Ö et al., 2018), among others. Resistance to T-DM1 has become a significant clinical challenge (Hunter et al., 2020). Currently, several clinical trials of T-DM1 combined with other drugs to delay disease progression in HER2-positive patients are underway, such as NCT02657343, which is studying the combination of CDK4/6 and T-DM1.

According to existing clinical research data, T-DXd has a relatively broad application range in the treatment of breast cancer. Whether it's HER2 low-expression breast cancer patients or HER2-positive breast cancer patients, T-DXd has shown certain therapeutic effects. This implies that T-DXd may become an essential choice for future breast cancer treatment, especially for those HER2-positive and HER2 low-expression patients who have poor outcomes with traditional treatments. However, ILD/pneumonia is a particular concern in the T-DXd treatment of breast cancer, a disease that can lead to pulmonary fibrosis and respiratory dysfunction, which can pose a threat to a patient’s life if not diagnosed and treated in a timely fashion (Abuhelwa et al., 2022; Powell et al., 2022). Currently, ILD is mainly treated by dose reduction, discontinuation of therapy, corticosteroids and supportive care, and the risk factors and underlying pathophysiology of T-DXd-induced ILD/pneumonia need to be further explored.

Another point of interest is that HER2 is widely regarded as the core predictive biomarker for HER2-targeted therapy. For antibody-drug conjugates, such as T-DM1, HER2 not only acts as a receptor but also involves effectively transporting the drug into cells. Therefore, the biomarker mechanism of ADC may include more complex intracellular drug handling links. RAB5A has been recognized as a potential predictive biomarker of response to T-DM1 therapy, and proteins involved in endoplasmic reticulum transport have also been identified as promising predictive biomarkers for ADC treatment strategies, according to scholar Olav Engebraaten, who tested whether clinical response to T-DM1, measured as the pCR, correlates with RAB5A RNA expression, as measured by the I-SPY2 clinical trial (NCT01042379), as a validation experiment. Whether clinical response to T-DM1, as measured by pCR, correlates with RAB5A RNA expression, the study clearly showed a significant correlation between RAB5A expression and breast cancer patients treated with T-DM1 (*p* = 0.01, LR test), which was additionally corroborated by the results of the KAMILLA clinical trial (NCT01702571) (Engebraaten et al., 2021; Wolf et al., 2022).

TNBC is still mainly treated with systemic treatments such as anthracyclines, taxanes, cyclophosphamide, and platinum (Won and Spruck, 2020). However, monotherapy has poor efficacy, combination therapy is prone to drug resistance, the prognosis is poor, and recurrent or refractory mTNBC is mostly palliative and aims to improve the patient’s quality of life (Li et al., 2019). From the completed clinical trials, SG has shown good efficacy in some heavily pretreated advanced mTNBCs. Trials such as NCT05382286 and NCT05382299 are evaluating the efficacy of SG in some untreated mTNBCs or early TNBCs. For HR+/HER2-metastatic breast cancer patients, several trials have shown that endocrine combined with CDK4/6 inhibitors can improve the patient’s OS(148). However, endocrine resistance is still severe in subsequent treatments, and SG has been found to be effective in treating HR+/HER2-metastatic breast cancer that has received at least one endocrine treatment or chemotherapy in the IMMU-132-01, TROPiCS-02 trials. The treatment of other types of breast cancer, such as HER2+, early breast cancer, etc., with SG remains to be seen. In general, the treatment of tricky TNBC with SG is promising. However, the cost-effectiveness of SG in treating breast cancer needs to be examined. An economic evaluation of SG in China and the United States showed that in China, SG added an extra 0.35 quality-adjusted life years (QALY), at a cost of ¥2257842. The incremental cost-effectiveness ratio (ICER) is ¥6375856 ($924037)/QALY. In the United States, SG produced the same additional QALY, but the extra cost was $175,393, with an ICER of $494,479/QALY, which has a lower cost-effectiveness ratio compared to chemotherapy drugs (Chen et al., 2021). In addition, there is still little real-world data on SG treatment for breast cancer, and pharmacoeconomic evaluations, other indications, drug interactions, biomarkers, etc., need further research and exploration. Although SG has been approved for mTNBC in several countries, the clinical trials that have confirmed the therapeutic effect of SG, such as the IMMU-132-01 trial, ASCENT trial, EVER-132–001 trial (as of 6 August 2021, 80 Chinese female patients were included), have included fewer Asian cases. Therefore, the therapeutic effect of SG in different races also needs to be explored.
